# A small-diameter vascular graft immobilized peptides for capturing endothelial colony-forming cells

**DOI:** 10.3389/fbioe.2023.1154986

**Published:** 2023-04-10

**Authors:** Yaqi Tang, Lu Yin, Shuai Gao, Xiaojing Long, Zhanhui Du, Yingchao Zhou, Shuiyan Zhao, Yue Cao, Silin Pan

**Affiliations:** ^1^ Heart Center, Qingdao Women and Children’s Hospital, Qingdao University, Qingdao, China; ^2^ College of Material, Chemistry and Chemical Engineering, Hangzhou Normal University, Hangzhou, China; ^3^ State Key Laboratory of Bio-fibers and Eco-textiles, Qingdao University, Qingdao, China

**Keywords:** rapid endothelialization, peptide immobilization, *in situ* differentiation, SDVDs, endothelial colony-forming cells

## Abstract

Combining synthetic polymers and biomacromolecules prevents the occurrence of thrombogenicity and intimal hyperplasia in small-diameter vascular grafts (SDVGs). In the present study, an electrospinning poly (L)-lactic acid (PLLA) bilayered scaffold is developed to prevent thrombosis after implantation by promoting the capture and differentiation of endothelial colony-forming cells (ECFCs). The scaffold consists of an outer PLLA scaffold and an inner porous PLLA biomimetic membrane combined with heparin (Hep), peptide Gly-Gly-Gly-Arg-Glu-Asp-Val (GGG-REDV), and vascular endothelial growth factor (VEGF). Attenuated total reflection Fourier transform infrared (ATR-FTIR) spectroscopy, X-ray photoelectron spectroscopy (XPS), and contact angle goniometry were performed to determine successful synthesis. The tensile strength of the outer layer was obtained using the recorded stress/strain curves, and hemocompatibility was evaluated using the blood clotting test. The proliferation, function, and differentiation properties of ECFCs were measured on various surfaces. Scanning electronic microscopy (SEM) was used to observe the morphology of ECFCs on the surface. The outer layer of scaffolds exhibited a similar strain and stress performance as the human saphenous vein via the tensile experiment. The contact angle decreased continuously until it reached 56° after REDV/VEGF modification, and SEM images of platelet adhesion showed a better hemocompatibility surface after modification. The ECFCs were captured using the REDV + VEGF + surface successfully under flow conditions. The expression of mature ECs was constantly increased with the culture of ECFCs on REDV + VEGF + surfaces. SEM images showed that the ECFCs captured by the REDV + VEGF + surface formed capillary-like structures after 4 weeks of culture. The SDVGs modified by REDV combined with VEGF promoted ECFC capture and rapid differentiation into ECs, forming capillary-like structures *in vitro*. The bilayered SDVGs could be used as vascular devices that achieved a high patency rate and rapid re-endothelialization.

## 1 Introduction

Cardiovascular diseases (CVDs) are a group of disorders that are the most lethal non-communicable diseases worldwide. They account for approximately 45% of all deaths in the European Union yearly. The high medical cost of CVDs brings a substantial economic burden on families and society (Timmis et al., 2018). An artificial vascular scaffold is a potential solution for treating severe CVDs (Green and Elisseeff, 2016). Synthetic vascular grafts fabricated using expanded polytetrafluoroethylene (ePTFE) are available and provide clinical efficiency (Pagani, 2019). However, these grafts have a low patency rate because of non-compliance, thrombogenic surfaces, and a tendency to induce intimal hyperplasia (de Mel et al., 2008), particularly in small-diameter vascular grafts (SDVGs) (Mi et al., 2019). Hence, it is extremely urgent to fabricate SDVGs with good mechanical properties and high patency rates.

A tissue-engineered vessel promoting *in situ* endothelialization in grafts would significantly benefit the patency rate (Wei et al., 2022). One strategy in endothelialization is to present endothelium-derived macromolecules or their cell-interacting domains onto the lumen surfaces of vascular scaffolds. This mimics the features of the extracellular matrix (ECM) to assist endothelial cell (EC)/endothelial progenitor cell (EPC) capture (Mp and Ja, 2005; Lehtinen et al., 2012; Su et al., 2017).

The peptide REDV (Arg–Glu–Asp–Val), which can bind specifically to α4β1 integrin on EPCs, was previously demonstrated to reduce the EPC velocity while rolling, but not to support EPC adhesion (Seeto et al., 2013). However, Atsushi and others demonstrated that the REDV ligand captured endothelial colony-forming cells (ECFCs), a type of EPCs, in a sequence-specific manner under shear flow conditions (Mahara et al., 2021). In addition, endothelium-derived macromolecules such as vascular endothelial growth factor (VEGF) can mobilize ECFCs, which regulates angiogenesis and vascular maturation (Ferrara et al., 2003; Kutikhin et al., 2020; Melero-Martin, 2022). As a common surface modification agent of biological materials, heparin (Hep) is a component of the ECM and an anticoagulant linked to the scaffold surface (Lu et al., 2013). These biomolecules can mimic ECM features and promote *in situ* endothelialization (Sarkar et al., 2007; Ercolani et al., 2015).

Providing templates for cell culture in scaffolds is a popular method in tissue engineering. Compared with other strategies, electrospinning presents physical properties close to those of the ECM structure by generating polymer-based fibers with diameters on the nano- to micro-scale (Yang et al., 2016; Dziemidowicz et al., 2021). Ideal materials for artificial vascular scaffolds were sought to satisfy both excellent biocompatibility and favorable mechanics for clinical application. Poly (L)-lactic acid (PLLA) is widely explored in tissue engineering and drug-delivery systems because of its remarkable biodegradability and biocompatibility properties (Tyler et al., 2016). The application of electrospinning PLLA to fabricate artificial vascular scaffolds has been recognized in many studies (Song et al., 2021; Baek et al., 2022). Moreover, we presented a strategy to fabricate porous PLLA fabrics with both micro- and nano-pores using a combination of electrospinning PLLA/polyethylene oxide (PEO) blend solution and water etching of PEO in the previous work (Zhao et al., 2018). The porous surface creates a higher surface area and cytocompatibility and has increased potential for loading growth factors (Savio et al., 2018).

Therefore, we fabricated an electrospinning PLLA bilayered scaffold consisting of the outer layer PLLA scaffold for support and the inner porous PLLA fabrics, which combined with REDV, VEGF, and Hep prevent blood coagulation as well as promote the capture and differentiation of ECFCs and allow quick vascular re-endothelialization.

## 2 Materials and methods

### 2.1 Preparation of the outer layer PLLA membrane and inner porous PLLA fabrics

PLLA (3001 D, *Mn* = 8.9 × 10^4^ g/mol, *Mw/Mn* = 2.0, Nature Work, United States) was dissolved in N, N-dimethylformamide (DMF) and dichloromethane (DCM) (Hangzhou Shuanglin Chemical Reagent Co., Ltd., China). The solutions were electrospun from a 5-mL syringe with a blunt-ended needle (diameter = 0.6 mm) under 15 kV high voltage and 20 cm receiver distance. The injection rate was set at 0.1 mL/h, with fibers collected using a sheet of aluminum foil. The PLLA membrane was dried in a vacuum at 40°C for 6 h.

PLLA and PEO (9 wt%, Sigma-Aldrich, United States) were dissolved in chloroform (Hangzhou Shuanglin Chemical Reagent Co., Ltd., China) before electrospinning (ratio of PLLA/PEO = 1:1). The electrospinning process is conducted in the same manner as in the previous step, except the aluminum foil was replaced by a coaxial spinneret needle. The samples were immersed in deionized water at room temperature for 24 h to etch the PEO phase, and the etched samples were dried in vacuum at 40°C for 6 h.

### 2.2 Mechanical properties of the outer layer scaffold

Thermoforming was performed to fabricate the outer layer PLLA of the bilayered scaffold. Briefly, a piece of a membrane (500 × 500 mm^2^, 15 μm in thickness) was rolled up around a glass rod, close to, but not touching, a soldering iron at 140°C–180°C.

The membrane piece (15 × 15 mm^2^) was placed on a tensile testing machine (CMT6103, MTS, United States) equipped with a 10-N unit load (5 mm/min deformation rate) for tensile tests. Three membranes (as a group) were used for tensile testing based on the recorded stress/strain curves, and the tensile strength was obtained.

### 2.3 Preparation of the functional biomimetic membrane

**FIGURE 1:**
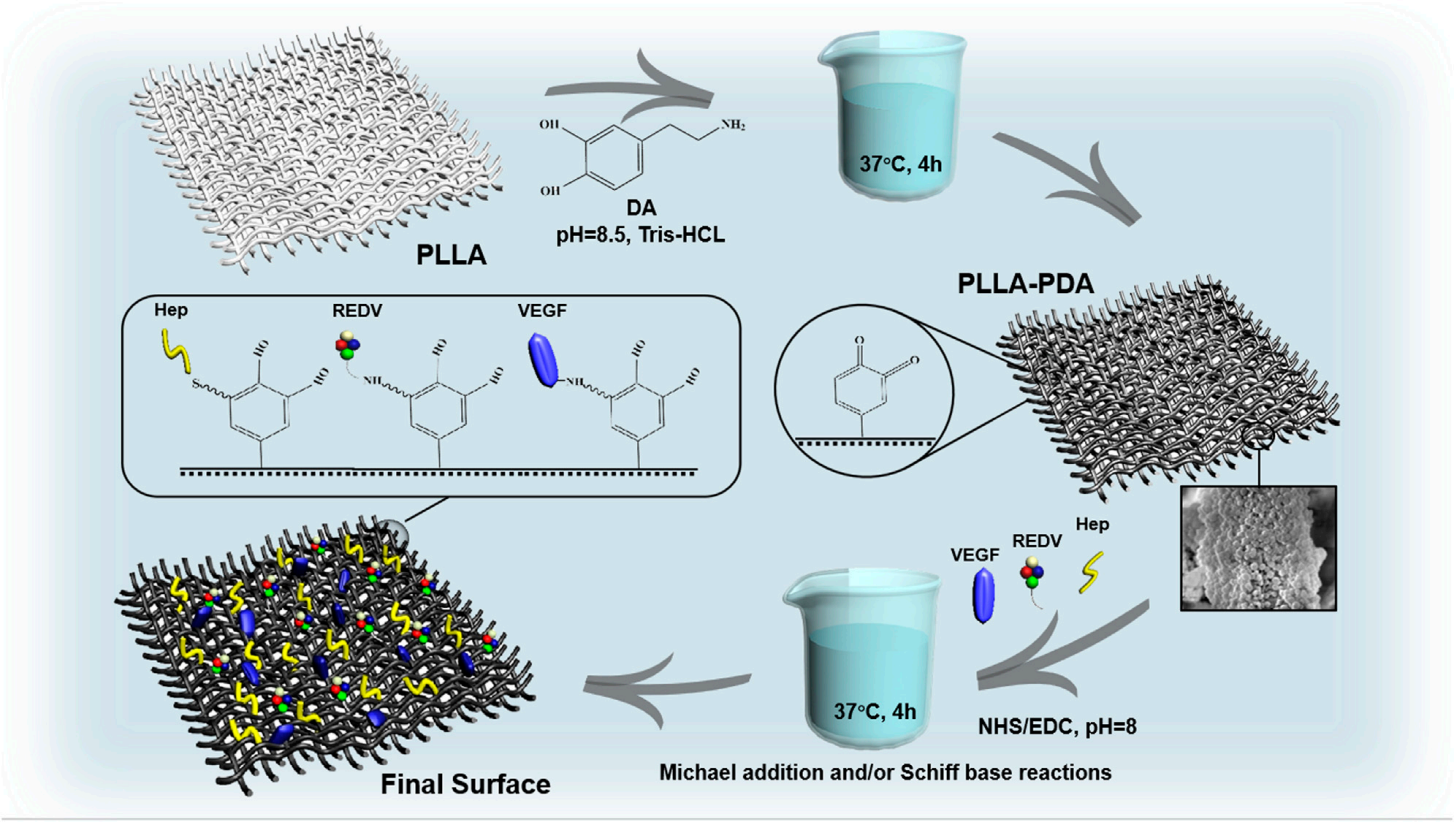
Preparation process of the biomimetic membrane involving Michael addition and/or Schiff base reactions under the catalysis of EDC/NHS.

Dopamine (DA) can be used in biomaterial surface modification methods because of its ease of use and low cost (Lee et al., 2007). The inner layer porous PLLA fabric was immersed in 3,4-dihydroxyphenylalanine (2 mg/mL) and dissolved in Tris-HCl buffer (10 mM, pH 8.5). Next, it was shaken on a rocker at 37°C for 4 h for polydopamine (PDA) coating before washing it with ddH_2_O to remove weakly bound PDA. In alkaline aqueous solutions, PDA contains amine and/or thiol groups on their surfaces via Michael addition and/or Schiff base reactions. This occurs during the catalysis of 1-(3-dimethylaminopropyl)-3-ethylcarbodiimide hydrochloride (EDC) and N-hydroxysuccinimide (NHS). Briefly, EDC (15 mg, N835594, MACKLIN, China) and NHS (15 mg, H6231, MACKLIN, China) were dissolved in phosphate-buffered saline (3 mL, PBS, Jiangsu KeyGEN BioTECH, China) with the mixture of heparin (Hep, H8060, Solarbio, China), peptides GGG-REDV (Nanjing TGpeptide Co., Ltd., China), and recombinant human VEGF165 protein (HZ1038, Proteintech, United States). The membrane surfaces were incubated in this solution at 37°C for 4 h before extensive washing with ddH_2_O and freeze-drying (Christ Alpha 1-2 LDplus, Christ, Germany) (Figure 1).

### 2.4 Characterization of the biomimetic membrane

To observe the change in hydrophilicity of the modified surface, the PLLA fiber mats were characterized using a contact angle goniometer (Theta, Biolin, Sweden). A piece of 1 × 1 cm^2^ mat was attached to a glass slide, a drop of ddH_2_O was placed on the surface of the fiber mats, and the contact angle was measured after 1.5 s. The experiments were repeated three times, and the data were expressed as the mean ± SD. Attenuated total reflection Fourier transform infrared (ATR-FTIR, Nicolet iS50, Thermo Fisher Scientific, United States) spectroscopy was performed to analyze the surface chemical structures of different mats. The spectra were measured in a wave number range from 4,000 cm^−1^–400 cm^−1^.

X-ray photoelectron spectroscopy (XPS) (ESCALAB Xi+, Thermo Fisher Scientific, United States) characterized the composition of various modified surfaces with a monochromated Al/Ka as an X-ray source. The surface spectra were collected from 0 to 1,400 eV, and the C1s and O1s regions were provided as higher-solution spectra. The concentration ratio of the C-O and C=O to C-C on the surfaces was determined by peak-area ratios using Avantage software.

The thermogravimetric analysis was performed to determine the amount of coating on the surface (TGA5500, TG, United States). For the control analysis, the PLLA, PDA, Hep, and GGG-REDV were held at 25°C for 5 min, after which the temperature was increased to 950°C at a heating rate of 10 °C/min. Nitrogen was used as the purging gas at a rate of 40 mL/min. The TGA curves of control samples, PLLA-PDA-REDV, PLLA-PDA-Hep, and final surface (PLLA-PDA-Hep-REDV-VEGF) were measured. The DTG curve was generated using the “1st derivative” option in Origin software.

### 2.5 Platelet adhesion and the blood clotting test

The membrane was added to the mixed aqueous solution of PDA/Hep (concentration ratios of 0.5:3, 0.5:5, 1:1, 1:3, 1:4, 2:2, and 2:3) to determine the best concentration of covalent binding for anti-platelet adhesion. First, whole blood from healthy volunteers was collected in tubes with ethylene glycol tetraacetic acid and then centrifuged at 2000 rpm for 15 min to obtain the serum, which was extracted for centrifuging again for the remaining liquid (1/4) in the platelet-rich plasma (PRP). The mats with a different PDA:Hep concentration ratio were incubated at 37°C (80 rpm) with 10 μL PRP added to 1 mL PBS and gently shaken to remove loosely adhered platelets after 2 h. The mats were fixed with glutaraldehyde for half an hour and then dried with a freeze-dryer. The adherent platelets were examined using scanning electron microscopy (SEM, JSM-6390LV, JEOL, Japan). After determining the proper concentration ratio of PDA:Hep, we prepared the mats with Hep+, R+, V+, and both R+ and V+ (R+V+) to test the influence of REDV and VEGF on platelet adhesion. Statistical analysis was performed based on three images randomly selected from different fields for each sample.

The 100 μL whole blood from healthy volunteers was directly added onto the surface of PLLA, PDA+, and Hep+ (1 × 1 cm^2^) for a dynamic blood clotting test. After the mats were incubated at 37°C for 10, 20, 30, and 40 min, 500 μL of ddH_2_O was added to induce hemolysis. The hemoglobin from erythrocytes, which was still not coagulated, was semi-quantified by measuring hemoglobin in uncoagulated erythrocytes in the blood (O.D. = 540 nm) using a microplate reader. (Synergy Neo2, BioTek, United States).

### 2.6 Protein adsorption test

Human serum albumin (HSA) and fibrinogen adsorption experiments were conducted under dynamic conditions. The HSA (2 mg/mL) and fibrinogen (1 mg/mL) were dissolved in PBS, respectively. The surfaces of PLLA, PDA+, and Hep+ (1 × 1 cm^2^) were incubated in a solution at 37°C (80 rpm) for 2 h. The amount of adsorbed protein was calculated from the concentration difference of the HSA/fibrinogen solution before and after adsorption, as determined using the BCA Protein Assay Reagent Kit (A53225, Thermo Fisher Scientific). A microplate reader was used to measure the adsorption (O.D. = 480 nm). Three tests were performed in parallel for each sample, and the data were expressed as mean ± SD.

### 2.7 Isolation of mononuclear cells and ECFCs culture

Peripheral blood mononuclear cells (PBMCs) were isolated from rats by peripheral blood lymphocyte separation fluid (LTS1083, Tianjin Haoyang Biological Manufacture Co., Ltd., China). This was carried out according to the manufacturer’s instructions and following the protocol modified by Kolbe et al., (2010). Briefly, the 25 cm^2^ culture flasks (356,484, Corning, United States) were coated with 2 μg/cm^2^ collagen (C8062, Solarbio, China) dissolved in 0.006 mg/mL acetic acid (A801295, MACKLIN, China). The isolated PBMCs (1 × 10^7^ in total) were resuspended in the EGM-2MV (CC-3202, Lonza, Switzerland) medium with 5% fetal calf serum (16140071, Gibco, United States) and 1% penicillin (10378016, Gibco, United States) and seeded into the collagen-coated flasks. The medium was changed daily to remove non-adherent PBMCs in the first 2 days, and this was changed approximately two–three times a week. After 1 week of culture, the PBMCs were dissociated with trypsin (15090046, Gibco, United States) and reseeded in flasks coated with 16 μg/cm^2^ of fibronectin (FN, F8180, Solarbio, China). Immunophenotyping and functional assays were performed after 4 weeks of culture. The cells were fixed in 4% paraformaldehyde for 30 min and then treated with 0.1% Triton X-100 (T8787, Sigma, Japan) for 15 min. Next, the cells were treated with 1% BSA (A9418, Sigma, Japan) for 30 min and incubated with anti-CD31 (ab119339, Abcam, 1:100, United States), CD45 (ab40763, Abcam, 1:100, United States), and CD133 (ab278053, Abcam, 1:100, United States) overnight at 4°C. After washing with PBS three times, cells were labeled with the corresponding secondary antibody, Alexa Fluor 647 anti-rabbit (ab7481, Abcam, 1:200, United States), for 1 h at room temperature. Finally, the cell nuclei were counterstained with 4’,6-diamidino-2-phenylindole (DAPI, ab285390, Abcam, United States) at a concentration of 10 μg/mL. Additionally, the acetylated low-density lipoprotein cholesterol (ac-LDL) and Ulex europaeus Agglutinin-1 (UEA-1) uptake by ECFC were assessed. Briefly, 1 ug/mL of DiI-labeled low-density lipoprotein (DiI-acLDL, MP6013, Shanghai MKBio Co., Ltd., China) was added to the cells and incubated at 37°C for 4 h. Then, 4% paraformaldehyde was added for 20 min and incubated for 1 h at 37°C with FITC-conjugated UEA (MP6308, Shanghai MKBio Co., Ltd., China) at a concentration of 5 μg/mL. The nuclei were counterstained with DAPI for confocal microscopy using an LSM 900&Axio Imager M2 (Carl Zeiss, Germany).

### 2.8 Specific ECFCs capture

To determine the appropriate surfaces of GGG-REDV capture, different mats of PLLA, Hep+, REDV+, VEGF+, and R+V+ were prepared for covalent binding to get the best capture effect of ECFC. The ECFCs cultured for 2 weeks were labeled with CM-Dil (40718ES50, YEASEN, China). Specifically, the medium was removed, and fresh medium with CM-Dil (1 ug/mL) was added and cultured for 30 min in a cell incubator (CCL-170T-8, ESCO, Singapore). After 1 day of culture, the cells were dissociated with trypsin and were resuspended in the medium on different surfaces for incubation on a shaker (TIPS300, Shanghai TIPS Biological Co., Ltd., China) at 80 rpm in a cell incubator. During the capture, the new medium was changed every 1 h to remove non-adherent cells (for 4 h). After that, the mats were washed with PBS and fixed with 4% paraformaldehyde. The morphology and numbers of the captured cell were examined using confocal microscopy (six pictures were randomly captured for statistical analysis).

To assess the capture ability more precisely, freshly isolated PBMCs were plated on the PLLA, Hep+, R+, V+, and R+V+ surfaces and maintained in a medium immediately after isolation. The medium was changed daily to remove non-adherent cells during the first 2 days. To verify whether the captured cells were CD133+ cells with the potential to differentiate into ECs, the PBMCs captured for 48 h were examined by flow cytometry (FCM). Specifically, a total of 100 µL of the medium containing ECFCs (3 × 10^5^) removed from the plates using trypsin and washed with PBS was taken for staining. This was carried out using conjugated mouse recombinant multiclonal CD133 primary antibodies (sc-365537, SCBT, United States, 1:500) at 4°C for 2 h, and then the cells were washed with PBS. Next, the conjugated secondary antibody Alexa Fluor 488 anti-mouse (ab150113, Abcam, United States, 1:200) was incubated at 4°C for 2 h, washed three times, and then resuspended in PBS. The samples were analyzed on an FCM (CytoFLEX LX, Beckman, United States) with FlowJo (v10.6.2) software.

### 2.9 Cell proliferation and function

Cell viability assay of the captured ECFCs on the mats (PLLA, Hep+, R+, V+, and R+ V+ surfaces) was performed. Briefly, after ECFCs were cultured for 5 weeks, they were captured on different engineered surfaces under shear stress and cultured for 1, 2, 3, and 4 days. The ECFCs were then dissociated with trypsin, resuspended in flasks, and cultured for 12 h to adhere to the cell wall. Then, the cells were stained using the Calcein AM/PI double stain kit (MX3012, Shanghai MKBio Co., Ltd., China). The Calcein AM was used to stain the live cells, while PI staining was used to stain dead cells. The results were examined using confocal microscopy (three pictures were randomly selected for statistical analysis).

A nitric oxide (NO) detection kit (S0021S, Beyotime, China) was utilized to determine NO quantity, following the instructions of the manufacturer, and the absorbance (O.D. = 540 nm) was measured using a microplate reader.

### 2.10 Co-culture of ECFCs and SMCs

To evaluate the effect of REDV transplantation on the proliferation of ECFCs, ECFCs cultured for 8 weeks were labeled with CM-Dil, while rat vascular smooth muscle cells (SMCs, kindly provided by Dr. Shuai Gao from Qingdao University, Qingdao, China) were labeled with CMFDA (40721ES50, YEASEN, China) and seeded on different surfaces at the same concentration (3 × 10^4^ cells). After 24 and 48 h of culture, the various mats were washed with PBS, fixed with 4% paraformaldehyde, and observed using confocal microscopy.

### 2.11 Observation of cell morphology by SEM

After 4 weeks of culture, the various mats that captured ECFCs were washed with PBS, fixed with 2.5% glutaraldehyde, and finally subjected to freeze-drying before being coated with gold and imaged by SEM for cell morphology. For the PBMCs, the mats were fixed with 2.5% glutaraldehyde and subjected to freeze-drying before being coated with gold and imaged by SEM for cell morphology after 2 weeks of culture.

### 2.12 Gene expression

Real-time quantitative polymerase chain reaction (RT-qPCR) analysis was carried out to determine the gene expression of ECFCs and PBMCs. Briefly, the various surfaces were washed in ice-cold PBS and lysed with TRIzol (R401-01, Vazyme, China) for the subsequent total RNA isolation after the ECFCs/PBMCs were captured and cultured for 4 and 8 weeks. Then, the sequence amplification kit (R323, Vazyme, China) was used to apply the one-step mRNA reverse transcription. The obtained cDNA was adopted as the template for subsequent PCR amplification. The RT-qPCR reactions were performed using the CFX96 system (BioRad, United States) and the ChamQ Universal SYBR qPCR (Q711, Vazyme, China). The expression of the target gene was calculated according to the following equation:

$$Fold\;change=2^{\left(-\Delta \Delta Ct\right)}.$$

Reduced glyceraldehyde-phosphate dehydrogenase (GAPDH) expression was used as the reference gene. The primers used were designed by Primer Premier 5 (PREMIER Biosoft, Canada), and the details are given in Supplementary Table S1.

### 2.13 Statistical analysis

Three independent experiments were performed, each with three parallel samples, and the data are presented as mean ± SD. The inter-group comparison was determined by one-way analysis of variance (ANOVA), and *p* < 0.05 was considered statistically significant.

## 3 Results

### 3.1 The morphology and mechanical properties of the scaffold

**FIGURE 2:**
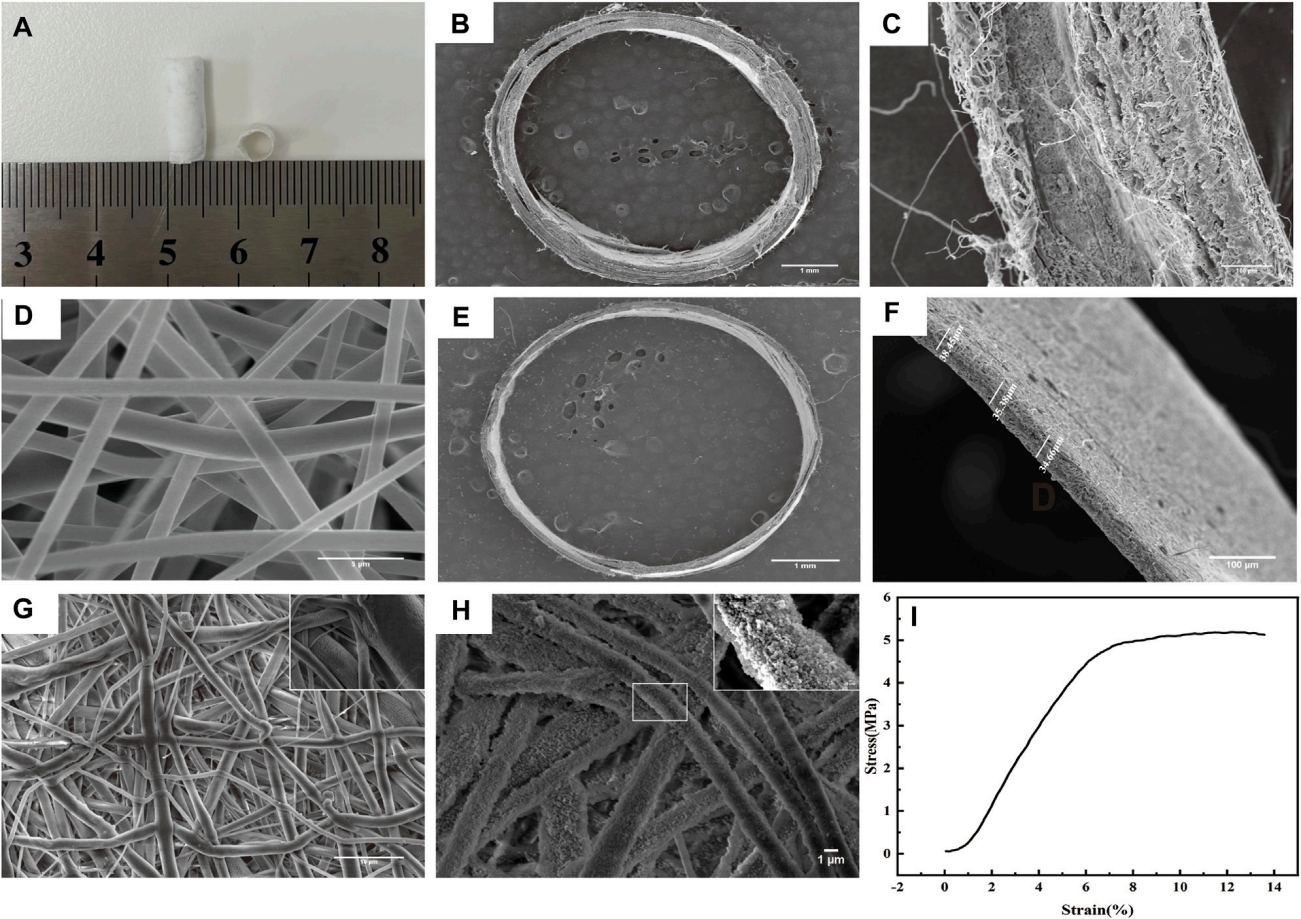
Morphology and mechanical properties of the scaffold. **(A)** Morphology of the scaffold. **(B-C)** Display of the double-layer structure of the scaffold by SEM. **(D)** Morphology of the outer layer of electrospun PLLA observed by SEM. **(E–F)** Average thickness of the inner layer was 36.16 ± 2.01 μm. **(G)** Morphology of the porous PLLA fabrics observed by SEM. **(H)** Morphology of PLLA modified with PDA observed by SEM. **(I)** Tensile strength of the outer layer scaffold fabricated by electrostatic spinning.

The unaided viewing appearance of the scaffold is shown in Figure 2A. The obvious double-layer structure of the scaffold is shown in Figures 2B, C. The microstructure of the outer layer of electrospun PLLA is shown in Figure 2D. The porous PLLA fabrics that etched the PEO phase possessed interconnected pores with randomly distributed fibers approximately 10 μm in diameter on average (Figure 2G). After modification by PDA, the fibers presented a rougher surface with evenly visible convex particles, indicating the presence of PDA coating on the PLLA (Figure 2H). The average thickness of the inner layer was 36.16 ± 2.01 μm (Figures 2E, F), which agrees with the native vascular intima size. The tensile experiment results showed excellent elongation performance of the outer layer fibrous membranes (13.38%) (Figure 2I).

### 3.2 Surface characterization of the inner fibrous membrane

**FIGURE 3:**
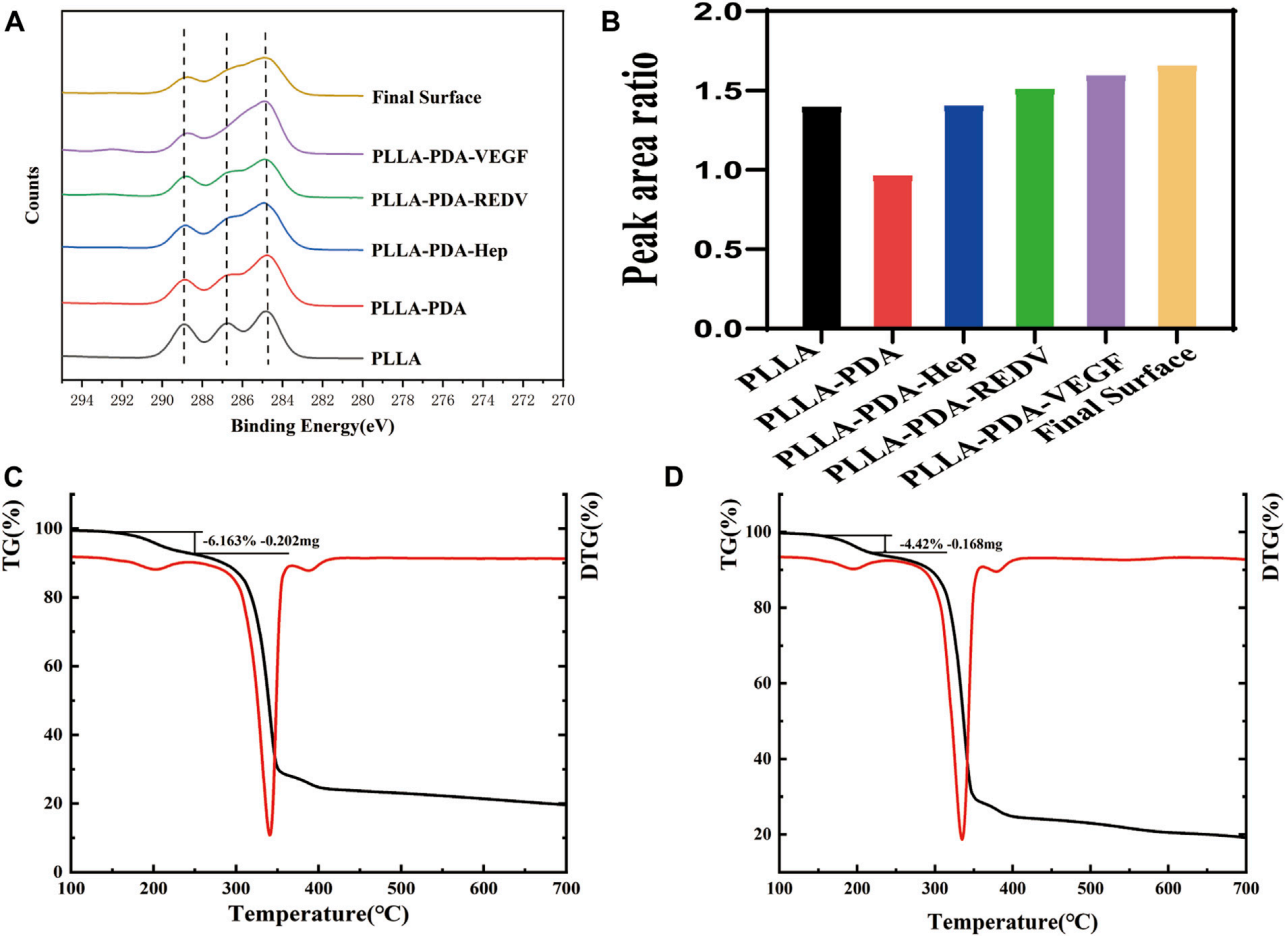
**(A-B)** The O/C atomic ratio composition of different surfaces represented by XPS. **(C)** Weight loss value of the PLLA–PDA–Hep surface analyzed by TGA and DTG curves. **(D)** Weight loss value of the PLLA–PDA–GGG-REDV surface analyzed by TGA and DTG curves.

The surface elemental composition in various surfaces was further investigated by XPS. The high-resolution C1s spectrum for PLLA showed three peaks at 284.4, 286.3, and 288.5 eV, representing the C-C/C-H, C-O, and O-C=O groups, respectively (Supplementary Figure S1). The concentration ratio of the C-O and C=O to C-C on the surfaces was determined by peak-area ratios using Avantage software. The ratio of the C-O and C=O to C-C on the surface was 1.39, and the ratio decreased significantly to 0.96 with the attachment of PDA. After Hep modification, the ratio increased to 1.40. The ratio of C-O and C=O to C-C was 1.50 with REDV modification. After VEGF modification, the ratio reached 1.59. The ratio of the final engineered surface (PLLA-PDA-Hep-REDV-VEGF) was 1.65 (Figures 3A, B). The result was consistent with the chemical formula of the various surface components (Supplementary Figure S1).

The TGA curves of different surfaces were obtained after thermogravimetric analysis. The DTG curve was generated using the “1st derivative” option in Analyzer software. The TGA and DTG curves show the decomposition of the surface coating and the amount remaining after measurement (Figures 3C, D). The DTG curve was used to draw a frame around the decomposition event and associate the frame to the appropriate position in the TGA curve. The weight loss value of GGG-REDV was 4.42% (0.168 mg), and that of Hep was 6.163% (0.202 mg). The decomposition temperature of each coating is shown in Supplementary Figure S2.

**FIGURE 4:**
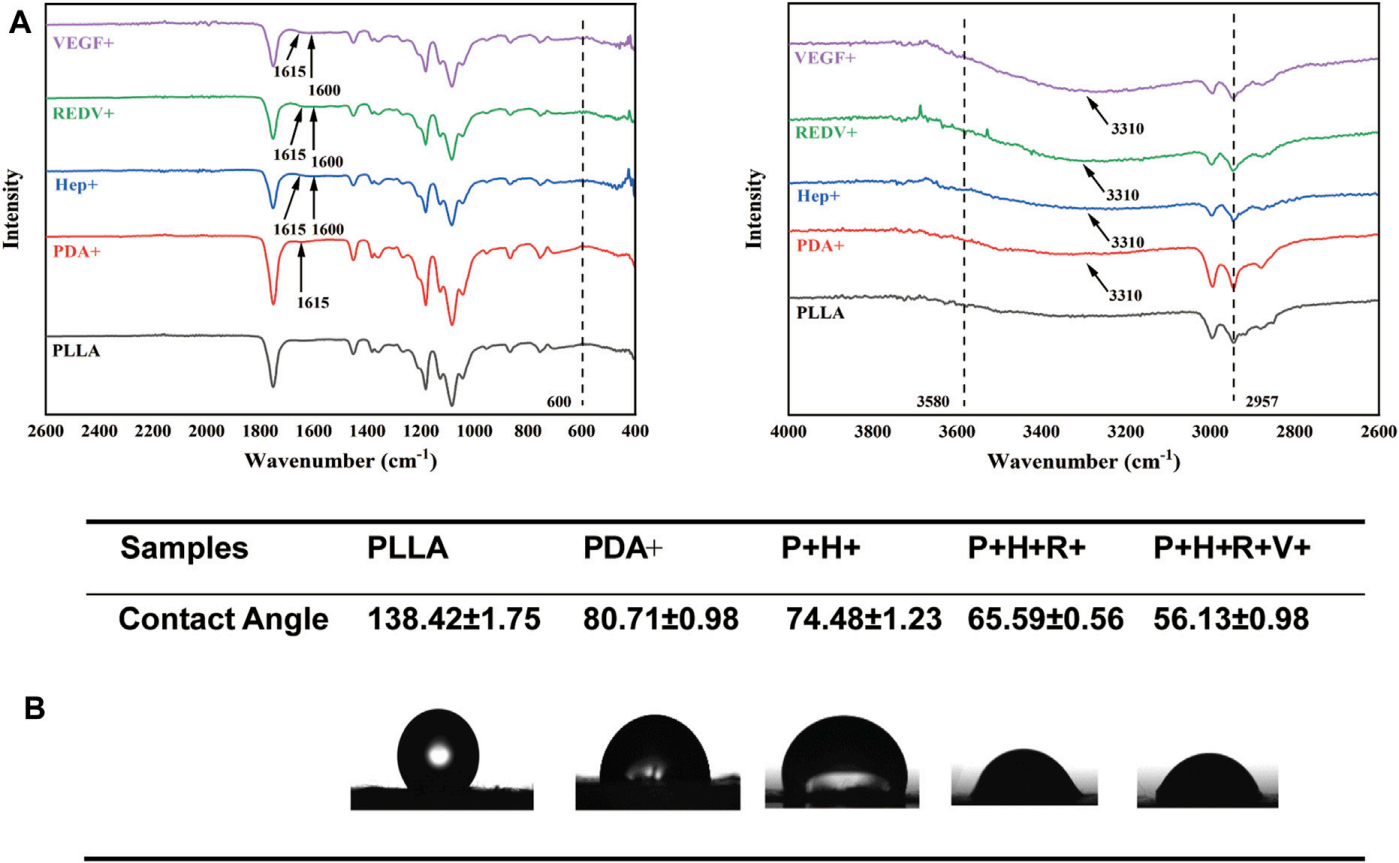
**(A)** Surface chemical structures of PLLA, PDA+, Hep+, R+, and R+V+ evaluated by ATR-FTIR. **(B)** Contact angle change of PLLA, PDA+, Hep+, R+, and R+V+ surfaces.

The surface chemical structures of various surfaces were analyzed by ATR-FTIR spectroscopy (Figure 4A). The bands at approximately 3,580 cm^−1^ and 2,957 cm^−1^ were attributed to the stretching of carbonyl and O-H asymmetric vibration of PLLA, respectively. The absorption peak for PLLA-PDA at 1,615 cm^−1^ was ascribed to the overlap of C=C stretching vibration in the aromatic ring and N-H bending vibration. After heparin modification, the absorption peak at 1,600 cm^−1^ was ascribed to the origin from the stretching vibrations of S=O bonds, indicating the successful grafting of heparin. Compared with the PLLA fiber mats, new absorption peaks at 3,300 cm^−1^ and 3,600 cm^−1^ appeared after being modified with Hep, REDV, and VEGF by Michael addition or Schiff base reaction (Kang et al., 2011). The hydrophilicity of the material noticeably increased with modification. Figure 4B shows that the PLLA mats demonstrated strong hydrophobicity, while the contact angle decreased continuously until it reached 56.13° after REDV/VEGF modification.

### 3.3 Hemocompatibility of the engineered surfaces

#### 3.3.1 Platelet adherent test

To determine the anti-platelet adherent effect of the biomimetic membrane, we incubated different concentrations of PDA/Hep with the PLLA. The results showed that the platelets were entangled with PDA particles on the fibers when the concentration of PDA was less than 1 mg/mL. On the contrary, when the concentration of PDA exceeds 2 mg/mL, the distribution of PDA particles on the fibers was uniform.

**FIGURE 5:**
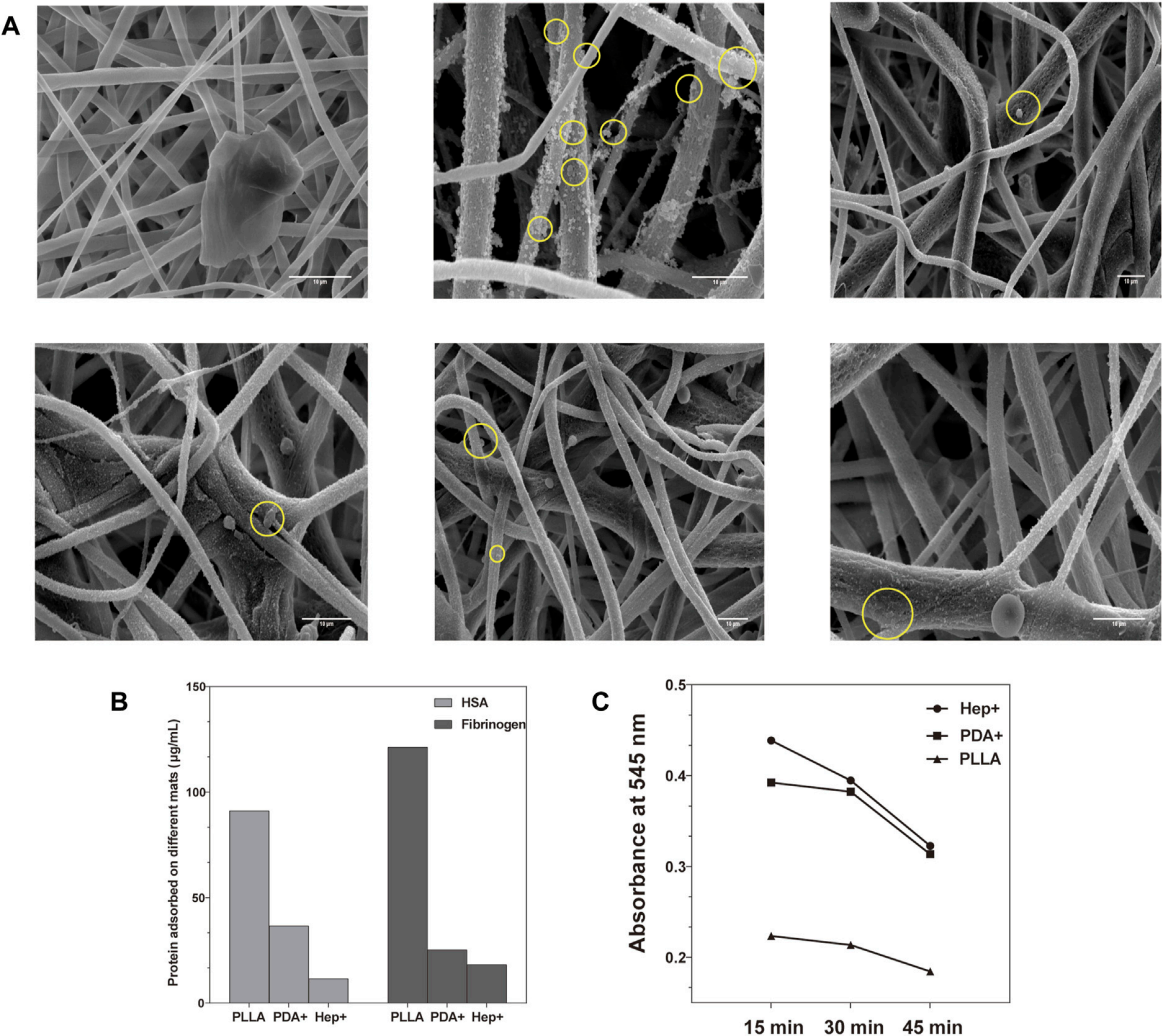
**(A)** Platelets spread into irregular shapes with pseudopodia on PDA surfaces while appearing in a round shape with little deformation on other surfaces evaluated by SEM. **(B)** Adhesion extent of non-specific albumin and fibrinogen on different modified surfaces. **(C)** Blood clotting speed was evaluated on different modified surfaces.

The platelets were spread into irregular shapes with pseudopodia on the surface in low (<2 mg/mL) heparin concentrations (Supplementary Figure S3). However, on the surface with a higher heparin concentration (>2 mg/mL), the platelet adhesion appeared round with slight deformation or pseudopodia. The platelet adhesion on the final function surface was observed by SEM (Figure 5A). As a result of the high hydrophobicity and low cytocompatibility of PLLA, a few platelets were observed to adhere to the unmodified PLLA surface. However, the platelets and PDA were adhered on the surface modified by PDA without Hep. After being modified by REDV and VEGF on the Hep + surface, platelet adhesion was lower compared with that of the PDA + but higher than that on Hep + only.

#### 3.3.2 Protein adsorption test

The adhesion of HSA and fibrinogen on different surfaces was observed by the absorbance at 480 nm (O.D.) (Figure 5B). Compared with the PLLA surface, the modified PLLA surface had lower HSA and fibrinogen adsorption (*p* < 0.05), especially in the Hep + surface (11.54 ± 0.56 μg/mL, 18.20 ± 0.36 μg/mL). The phenomenon may be attributed to the increased hydrophilicity and electrostatic repulsion between the negatively-charged heparin and HSA (Cornelius et al., 2003; Brash et al., 2019).

#### 3.3.3 Blood clotting test

The static clotting test showed that the blood clotting speed of modified surfaces was much lesser than that of the PLLA mats (Figure 5C). The O.D. value of the Hep + surface was significantly higher than that of the PLLA at 15, 30, and 45 min (*p* < 0.05). This demonstrated that blood clotting of the Hep + surface was lower than that of the PLLA surface.

These experiments demonstrate that the surface modified by Hep had good hemocompatibility. Despite being modified by REDV/VEGF, the Hep + surface influenced anti-platelet adhesion much better than the unmodified PLLA and modified by PDA surfaces.

#### 3.3.4 Extraction and cultivation of ECFCs

The PBMCs isolated from rat peripheral blood resulted in colony formation after 11 days of culture (Supplementary Figure S4A). The ECFC developed characteristics similar to HUVEC, where they internalized ac-LDL with the concurrent UEA binding (Supplementary Figure S4B). Our immunofluorescence results confirmed that ECFCs expressed endothelial specification in terms of CD31- and CD133-positive staining and CD45-negative staining (Supplementary Figure S4C). Both EPCs and ECs expressed VEGFR2, CD31, and vWF, while only EPC expressed CD133 (Khakoo and Finkel, 2005). After 8 weeks of culture, the expression of CD133 decreased gradually, while the expression of CD45 increased (Supplementary Figure S4D).

### 3.4 Modified surfaces promote ECFCs capture and differentiation into ECs

#### 3.4.1 Capture and proliferation of ECFCs

**FIGURE 6:**
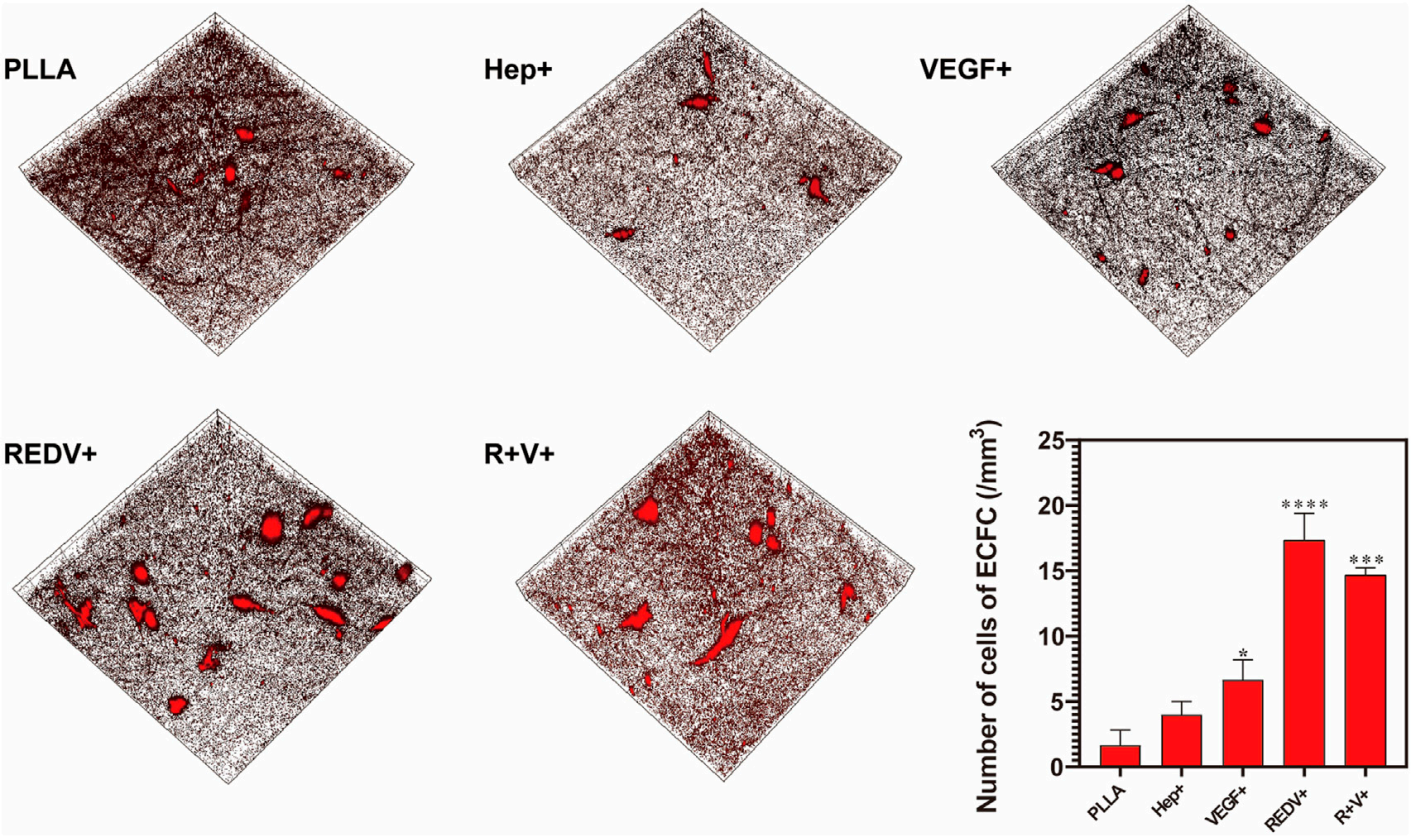
Fluorescence images of ECFC captured by the GGG-REDV successfully under flow conditions, with ×20 magnification, x = y = 399 μm, and Z_Pristine_ = 37 μm.

The fluorescent images confirmed that ECFCs were successfully captured by R+ and R+V+ surfaces under flow conditions. The number of ECFCs (17.33 cells/mm^3^) adherent on the R+ surface was significantly higher than that of others and of R + V+ (14.67 cells/mm^3^) as well (Figure 6) (*p* < 0.05). Cells on the R+V+ mats were typically elongated, and this forms a structural adaptation that optimizes EC function (Hahn and Schwartz, 2009). Although a few cells were deposited on PLLA (1.67 cells/mm^3^), Hep+ (4.00 cells/mm^3^), and V+ (6.67 cells/mm^3^) surfaces, the cells had no specific form and also performed worse in subsequent proliferation experiments.

**FIGURE 7:**
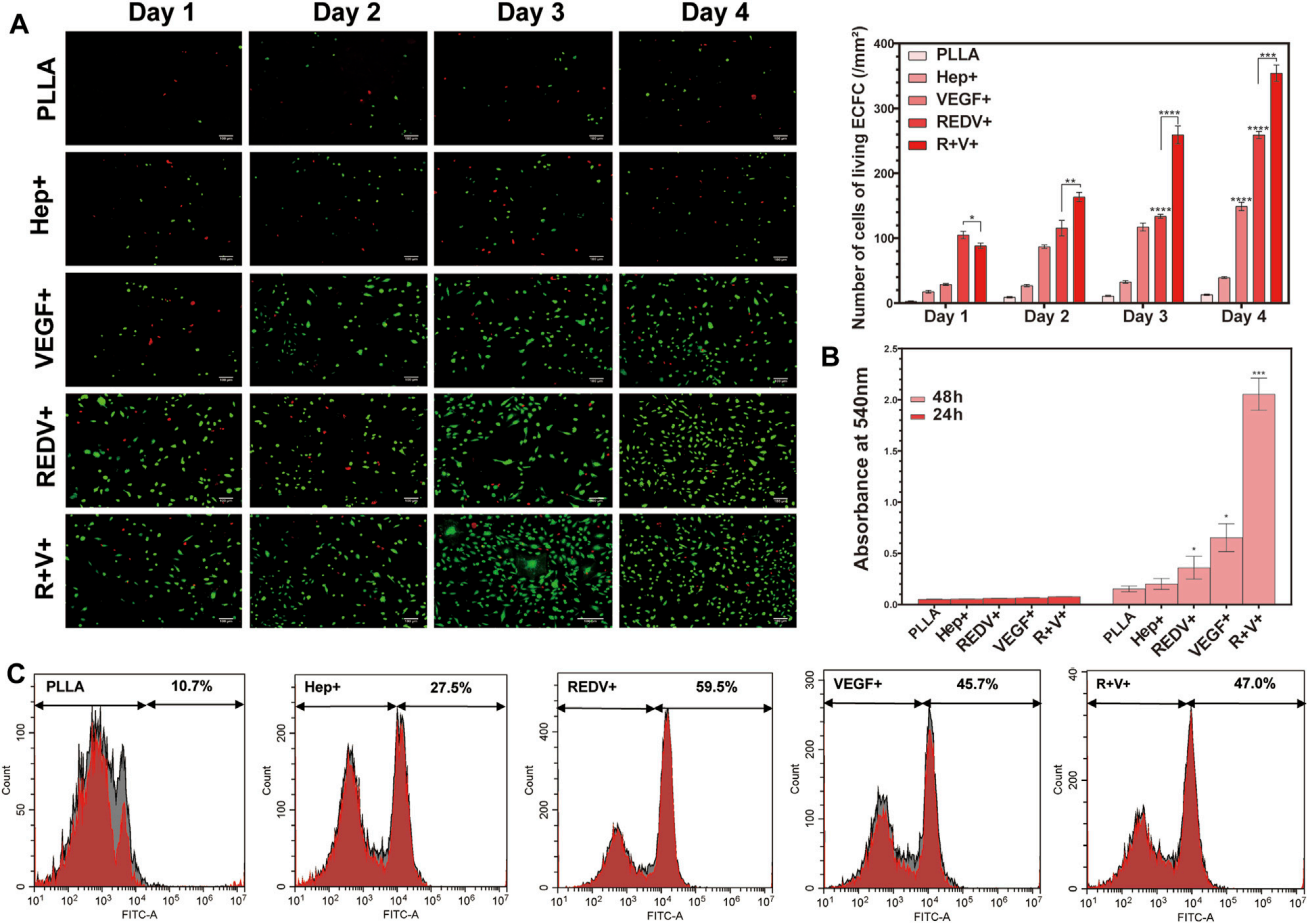
Proliferation of ECFCs on various surfaces. **(A)** AM/PI staining for cell proliferation and death at 1, 2, 3, and 4 days on various surfaces. **(B)** Release of NO along with the culture time. **(C)** Flow cytometry of CD133 positivity PBMCs captured by various surfaces.

Rapid re-endothelialization depends not only on the ECFC capture but also on the proliferation of ECFCs and their capacity for differentiation into ECs (Figure 7A). The number of living cells labeled by AM on the R+ surface was higher than that of others because of the superior performance of captured ECFCs on day 1 (*p* < 0.05). However, after 2 days of culture, the number of living cells labeled by AM on the R+V+ surface was significantly higher than that on R+, V+, or other surfaces (*p* < 0.05) and remained higher over time.

#### 3.4.2 Endothelial function test

Nitric oxide (NO) release experiments showed that the O.D. value of ECFC on the V+ and R+V+ surfaces significantly increased after 48 h of culture (*p* < 0.05), while there was no significant difference between various surfaces when cultured for 24 h (Figure 7B). Additionally, there was no significant difference in NO release on Hep+ and R+ surfaces compared with that of PLLA surfaces regardless of experimental time.

#### 3.4.3 Capture of CD133+ PBMCs

The PBMCs were seeded on PLLA, Hep+, R+, V+, and R+V+ surfaces, and the medium was changed after 24 and 48 h of culture. Flow cytometry (FCM) was carried out to detect the specification in terms of CD133 in PBMCs. More than 59% of cells cultured on the R+ surface demonstrated CD133^+^ positive staining using the FITC channel of FCM, while 47% were captured on the R+V+ surface (Figure 7C). Surprisingly, the V+ surface also presented a high capture rate of CD133+ cells (45.7%). However, the surface modified by VEGF only performed worse at promoting ECFC proliferation and inhibiting SMC proliferation in subsequent experiments.

#### 3.4.4 Gene expression and growth morphology of cells

**FIGURE 8:**
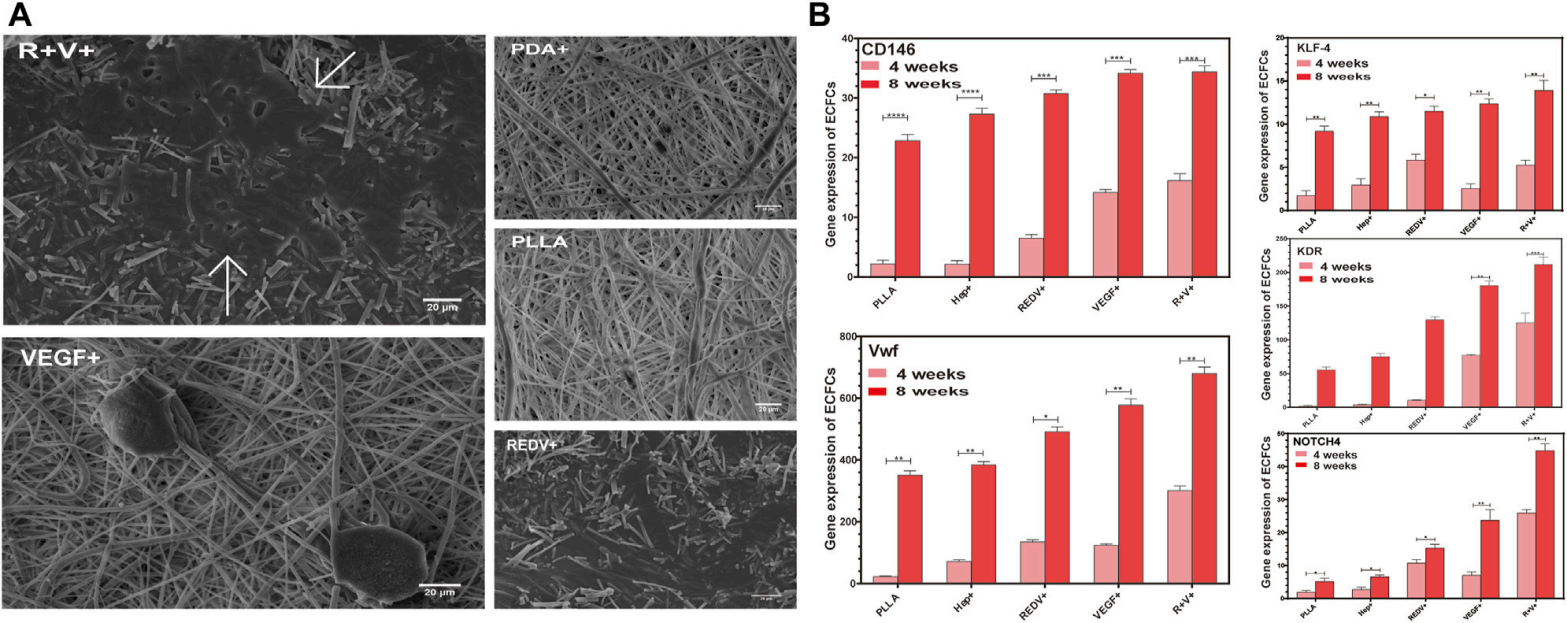
Gene expression and growth morphology of ECFCs. **(A)** Morphology of ECFCs captured by various surfaces and cultures for 4 weeks evaluated by SEM. **(B)** Endothelial signature gene expression of ECFCs after being captured on PLLA, Hep+, R+, V+, and R+V+ surfaces for 4 weeks and 8 weeks.

The ECFCs captured by various surfaces were grown and passaged for 4 weeks, and then the morphology was examined by SEM. The results showed that the diffusion area of ECFCs on the R+ surface was more significant than that on V+ or Hep + surfaces (Figure 8A), while cells on the R+V+ surface formed capillary-like structures after 4 weeks of culture, which suggested that the synergy of REDV and VEGF plays a vital role in promoting ECFC differentiation and EC tube-forming activity. In addition, RT-qPCR was performed for gene expression of ECFC culture on various surfaces. Endothelial signature gene expression of ECFCs before capture was performed as a control, and a value greater than 1 was considered to be the doubling of gene expression. The expression of vWF, KDR, NOTCH4, KLF4, and CD146 was constantly increased after 4 weeks and 8 weeks of culture of ECFCs on Hep+, R+, V+, and R+V+ surfaces (Figure 8B) (*p* < 0.05). ECFCs on the R+V+ surfaces expressed significantly higher pan-endothelial markers KDR (140.53-fold), key transcription factor KLF-4 (5.59-fold), CD146 (15.89-fold), vWF (311.04-fold), and arterial differentiation marker NOTCH4 (25.98-fold), which indicate that ECFCs on the peptide-modified R+V+ surface differentiated into ECs after capture for 4 weeks and presented a higher capability to assemble blood vessels as compared with other surfaces (Tzima et al., 2005).

On the PBMCs capture surface, the SEM images showed that the differentiation degree of PBMCs on the R+V+ surface was significantly higher after 2 weeks of culture compared with that on other surfaces (Supplementary Figure S5). Furthermore, PBMC culture for 8 weeks on the surface modified with REDV and VEGF overexpressed the mature EC markers CD146 (132.54-fold), vWF (282.08-fold), NOTCH4 (229.41-fold), and KLF-4 (576.48-fold) as compared with PBMCs culture isolated from blood (*p* < 0.05). The results indicated that the surface modified by R+V+ promotes PBMCs differentiation to ECFCs.

### 3.5 Co-culture of ECs and SMCs

**FIGURE 9:**
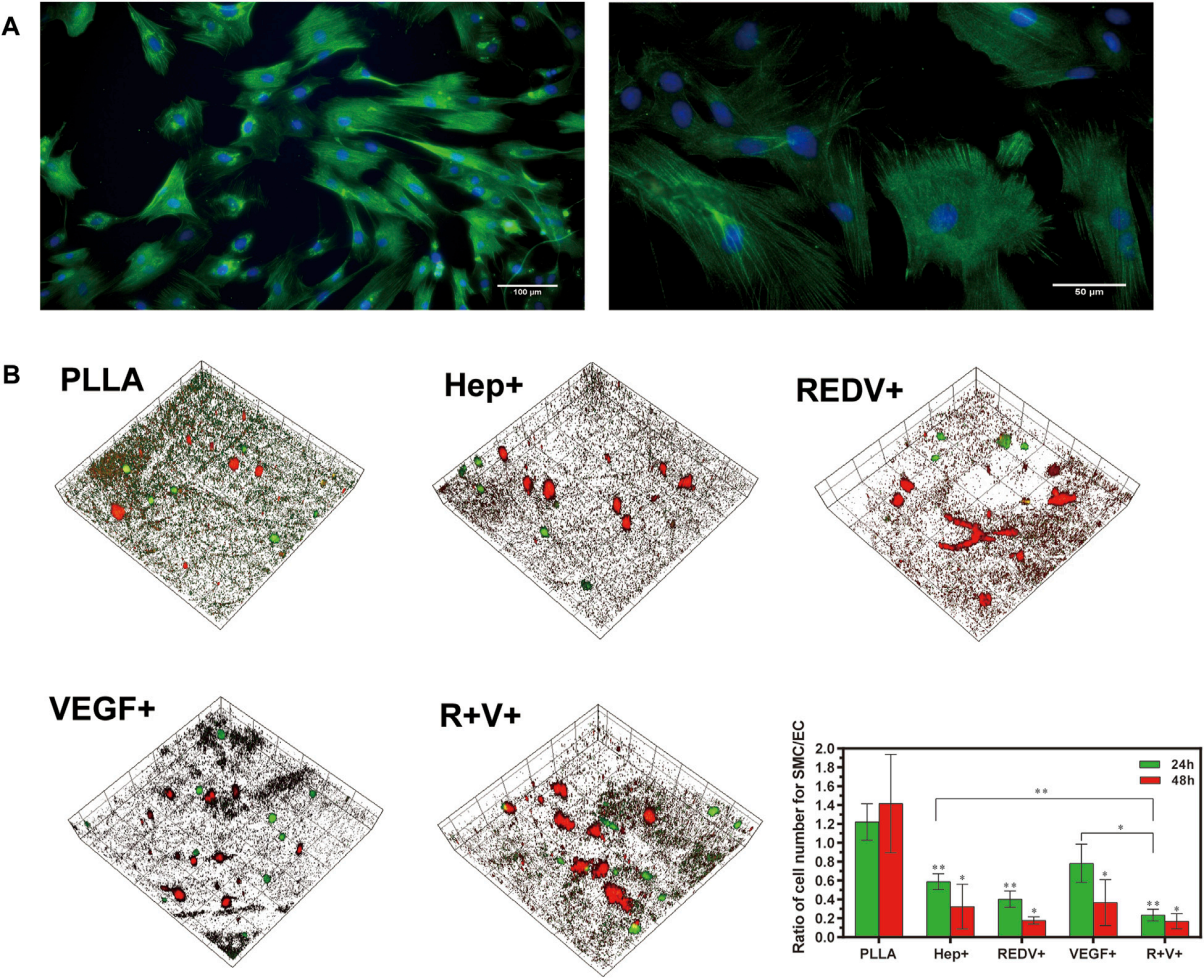
Co-culture of ECs and SMCs. **(A)** SMCs were stained with α-SMA and imaged by confocal microscopy. **(B)** 3D images of ECFCs and SMCs co-cultured at 24 and 48 h, with × 20 magnification, x = y = 399 μm, and Z_Pristine_ = 37 μm.

The rat vascular SMCs were stained with α-SMA and imaged by confocal microscopy (Figure 9A). To determine the proliferation selectivity of cells, an equal density of SMCs and ECs from ECFCs differentiation was seeded onto the PLLA, Hep+, R+, V+, and R+V+ surfaces. The morphology of the SMCs and ECs were evaluated using confocal microscopy after culture for 24 and 48 h. Figure 9B shows that a higher ratio of cell number for SMCs/ECs was obtained on PLLA (1.22, 1.42), in comparison to Hep+ (0.59, 0.32), R+ (0.40, 0.18), and R + V+ (0.23, 0.17) surfaces, at 24 and 48 h (*p* < 0.05). The SMCs exhibited minimal spreading and little proliferation on R+ surfaces at 48 h (0.17). The growth of SMCs was inhibited on the V+ surface and may result from the modification of PDA and Hep since there was no report about the inhibitory effect of VEGF on SMCs. The present study pointed out that the Hep–REDV–VEGF surfaces positively improved the proliferation of ECs and inhibited the SMCs.

## 4 Discussion

Autologous vessel grafts represent the gold standard for SDVGs (<6 mm) and are superior to synthetic grafts, but these vessels can induce acute thrombosis, neointimal hyperplasia, and accelerated atherosclerosis (Harskamp et al., 2013). Synthetic grafts are also available as a substitute for large-diameter vessels (>8 mm) and medium-diameter vessels (6–8 mm) (Brewster, 1997; Chlupáč et al., 2009). However, synthetic grafts are of limited utility because of poor patency rates in SDVGs. Therefore, tissue-engineered techniques that induce material endothelialization have been developed as alternative strategies, especially for SDVG development (Pashneh-Tala et al., 2016). For artificial vascular scaffold development, ECs can be cultured before implantation or captured in blood circulation to promote *in situ* endothelialization (Radke et al., 2018). Although considerable efforts have been made, the culture of ECs before implantation may cause severe restenosis (Radke et al., 2018). Although highly debated, the potential therapeutic action of EPCs has been demonstrated in basic research and clinical trials (Medina et al., 2017). However, the ambiguous nomenclature of EPCs and non-specific capture represent a critical barrier to *in situ* endothelialization. EPCs significantly contribute to new blood vessel formation by differentiating into ECs (Asahara et al., 1997). The late EPCs (ECFCs) are the only cell type with angiogenic potential (Medina et al., 2017). Nevertheless, most scholars agree that ECFCs express endothelial specification and CD45-negative staining (Case et al., 2007). The present study also confirmed that ECFCs derived from PBMCs expressed endothelial-specific markers like CD31-, CD133-positive staining, and CD4-5 negative staining (Supplementary Figure S4C). After culture for 8 weeks, ECFCs expressed CD133-negative staining while the expression of CD45 increased (Supplementary Figure S4D). In addition, we observed that the growth rate of ECFCs increased significantly after 8 weeks of culture, which differs from the first week (not shown in the results). These results were consistent with the generally recognized view that ECFCs can be differentiated into ECs after 8 weeks of culture (Li et al., 2017, *p*. 43).

Immobilization of biomolecules (including anti-CD34, anti-VEGFR2, and DNA aptamers) is recognized to be of little value because of the non-specific ECFCs capture and eventual restenosis (van Beusekom et al., 2012; Qi et al., 2015; Deng et al., 2017; Duan et al., 2019). Recently, there has been an increased focus on ECM peptide sequences (such as REDV, GRGDSP, and RGD) because of the potential to be used as vascular device coatings to recruit ECFCs (Ochsenhirt et al., 2006). The cell surface receptor a4 integrin plays a critical role in homing, engrafting, and maintaining hematopoietic progenitor cells in the bone marrow. Both α4β1 and α4β7 play critical roles in these processes via interactions with ligands expressed on the endothelial and stromal cell surfaces or in the extracellular matrix (Qin et al., 2006). Integrin α4β1 is prominent on mononuclear leukocytes, neutrophils, hematopoietic stem cells, and EPCs but platelets. It is a suitable candidate for application in vessels. It interacts with an alternatively spliced form of the extracellular matrix protein fibronectin. On the EPCs (Rodenberg and Pavalko, 2007), the peptide REDV, which can bind specifically to the α4β1 integrin, was previously demonstrated to reduce the ECFCs velocity while rolling, but not ECFCs firm adhesion (Seeto et al., 2013). However, researchers have found that combining different short peptides to REDV, such as Gly–Gly–Gly, could achieve the function of capturing ECFCs dynamically (Mahara et al., 2021; Tian et al., 2022).

The present study demonstrated bilayered SDVGs consisting of an inner layer co-immobilized with GGG-REDV and VEGF and an outer layer with a supporting function. The average thickness of the inner layer of the bilayered scaffold was 36.16 ± 2.01 μm, which agrees with the native vascular intima size. With the covalent binding mechanism provided by the PDA platform via the Michael addition reaction or Schiff base reaction, biomacromolecules were immobilized on the inner layer (proved by XPS and ATR-FTIR spectroscopy). The hydrophilicity of the inner layer was significantly improved after modifications that enhanced the cytocompatibility and reduced the adsorption of non-specific proteins (Paterlini et al., 2017). The fibrous outer layer presented a highly porous structure, which may have weakened the elongation performance (Song et al., 2021). However, the SDVG exhibited similar strain and stress performance with the human saphenous vein (Donovan et al., 1990).

Once the surface comes into contact with the blood, biomaterials will immediately obtain a layer of proteins, such as albumin, fibrinogen, and complement factors, forming a new interface between biomaterials and blood (Reynolds and Annich, 2011). The biomaterial surfaces are perceived and adhered to by immune cells, platelets, and red blood cells through this layer of proteins, which will result in coagulation, platelet adhesion, and activation (Jesmer and Wylie, 2020). The surface functionalized with Hep hindered the adsorption of protein platelets, which may have resulted from the contribution of the hydrophilic and negatively charged surface (Ye et al., 2012). Although REDV possesses low affinity to platelets, compared with the Hep + surface, the anticoagulant effect of REDV was slightly worse. However, considering the cell capture function, we can tolerate a slightly worse anticoagulant effect on the R+ surface.

Yuan et al. pointed out that the REDV and linker combinations could capture ECFCs under shear conditions (with a low platelet affinity) (Tian et al., 2022). By combining the linker Gly-Gly-Gly and REDV, we successfully achieved ECFCs capture in dynamic conditions, although a detailed structure of the integrin REDV binding site was not reported. A previous report has shown that when the REDV binds to the integrin, the metal cation in the *ß*-unit and the lipophilic group in the integrin come in contact with the hydrogen bonding acceptor in the REDV (Baiula et al., 2019). Even though the linker was conjugated to the N-terminal of the REDV peptide, the linker-conjugated REDV peptide still showed EPC specificity because of the two carboxyl groups on REDV for the binding domain in the integrin (Mahara et al., 2021).

In the present study, we successfully captured ECFCs under dynamic conditions with GGG-REDV and demonstrated rapid differentiation into ECs to form capillary-like structures *in vitro* (under the influence of REDV and VEGF) at 4 weeks (Figure 8A). NO release was similar to ECFCs proliferation (Figure 7B), which exhibited no significant change on the first day of culture. Typically, NO is released from the endothelium, and it regulates endothelial function. Additionally, it widens the blood vessel, allowing more blood to flow distally to that vessel. NO controls the internal diameter of blood vessels and promotes antiatherosclerosis and inhibition of platelet adhesion and leukocytes to the endothelium (Cooke and Tsao, 1994). It is worth noting that only surface immobilization with VEGF has promoted significantly enhanced NO release, which confirms the critical role of VEGF in promoting ECFCs function. Once bound to the ECM, VEGF can prolong VEGFR2 phosphorylation and subsequent signaling events on ECs (Anderson et al., 2009; Chen et al., 2010). Moreover, previous studies in ECs have demonstrated interactions and functional cross-talk between integrins and the VEGF/VEGFR2 system, including integrin-mediated adhesion to VEGF and VEGF-induced integrin activation (Byzova et al., 2000; Moulisová et al., 2017). In addition, the CD133-positive PBMCs can differentiate into ECFCs (Rossi et al., 2019). Flow cytometry showed that the R+ surfaces were more cell-selective and allowed CD133+ PBMC adhesion. This indicated that the REDV interaction with PBMCs might be similar to catch-bond behavior (Prezhdo and Pereverzev, 2009). Even though the R+ surface facilitates the capture of CD133+ PBMCs, the surface containing VEGF promotes differentiation into ECFCs and expresses higher mature EC markers than the R+ surface. Moreover, stable monolayer ECs and resting SMCs are required to maintain patency (Radke et al., 2018). Due to the complex competition of different cells in the body, ECs selectively promote and compete with SMCs (Brewster et al., 2006). The co-culture of ECs and SMCs demonstrated the proliferation selectivity of the inner layer. The REDV promotes attachment and mediates EC migration while inhibiting SMC adhesion (Plouffe et al., 2007). Figure 9B shows that the R+V+ surface shows a superior effect of proliferation selectivity compared with the PLLA, Hep+, and V+ surfaces. However, since Hep also promotes the inhibition of SMC proliferation (Ku and Park, 2010), there was no significant difference between R+ and Hep + surfaces concerning proliferation selectivity. The function of proliferation selectivity has been improved by including VEGF. Considering the undefined effects of VEGF on SMCs, we speculated that the inhibitory effects might contribute to rapid ECs proliferation.

## 5 Conclusion

In conclusion, the present study prepared a bilayered scaffold with the inner layer for a biomimetic membrane and the fibrous outer layer for braced force. The mechanical properties (strain and stress performance) of the outer layer scaffold were similar to those of the human saphenous vein. The inner layer surface immobilized with the peptide GGG-REDV and VEGF promoted ECFCs capture and rapid differentiation. This formed capillary-like structures *in vitro* and presented a positive effect on improving EC while inhibiting SMCs proliferation. Overall, the results supported the application of REDV to promote endothelialization in tissue-engineered SDVGs. The present study demonstrated that SDVGs fabricated using a facile method from electrospinning PLLA could form capillary-like structures on their surfaces via the capture and differentiation of endothelial colony-forming cells. This finding demonstrates that the scaffold has potential clinical applications. In further studies, we aim to perform animal experiments to verify the degradation rate and effect of *in situ* endothelialization. Additionally, we will conduct research *in vivo* and continue to improve the mechanical properties and re-endothelialization to allow for clinical translation.

## Data Availability

The original contributions presented in the study are included in the article/Supplementary Material, further inquiries can be directed to the corresponding author.

## Abbreviations

SDVGs, small-diameter vascular grafts; PLLA, poly(L)-lactic acid; Hep, heparin; REDV, Arg–Glu–Asp–Val; VEGF, vascular endothelial growth factor; ECFCs, endothelial colony-forming cells; ECs, endothelial cells; EPCs, endothelial progenitor cells; SMCs, smooth muscle cells; CVDs, cardiovascular diseases; NO, nitric oxide; ECM, extracellular matrix; SEM, scanning electronic microscopy; PEO, polyethylene oxide; PDA, polydopamine; DA, dopamine; EDC, 1-(3-dimethylaminopropyl)-3-ethylcarbodiimide hydrochloride; NHS, N-hydroxysuccinimide; FTIR, Fourier transform infrared; XPS, X-ray photoelectron spectroscopy; ANOVA, one-way analysis of variance; PRP, platelet-rich plasma; HAS, human serum albumin; PBMCs, peripheral blood mononuclear cells; FCM, flow cytometer; RT-qPCR, real-time quantitative polymerase chain reaction; GAPDH, glyceraldehyde-phosphate dehydrogenase.

## References

[B1] Anderson S. M. Chen T. T. Iruela-Arispe M. L. Segura T. (2009). The phosphorylation of vascular endothelial growth factor receptor-2 (VEGFR-2) by engineered surfaces with electrostatically or covalently immobilized VEGF. Biomaterials 30, 4618–4628. 10.1016/j.biomaterials.2009.05.030 19540581 PMC2826152

[B2] Asahara T. Murohara T. Sullivan A. Silver M. van der Zee R. Li T. (1997). Isolation of putative progenitor endothelial cells for angiogenesis. Science 275, 964–966. 10.1126/science.275.5302.964 9020076

[B3] Baek S.-W. Kim D.-S. Song D. H. Kim H. B. Lee S. Kim J. H. (2022). Reduced restenosis and enhanced re-endothelialization of functional biodegradable vascular scaffolds by everolimus and magnesium hydroxide. Biomater. Res. 26, 86. 10.1186/s40824-022-00334-x 36544178 PMC9768885

[B4] Baiula M. Spampinato S. Gentilucci L. Tolomelli A. (2019). Novel ligands targeting α4β1 integrin: Therapeutic applications and perspectives. Front. Chem. 7, 489. 10.3389/fchem.2019.00489 31338363 PMC6629825

[B5] Brash J. L. Horbett T. A. Latour R. A. Tengvall P. (2019). The blood compatibility challenge. Part 2: Protein adsorption phenomena governing blood reactivity. Acta Biomater. 94, 11–24. 10.1016/j.actbio.2019.06.022 31226477 PMC6642842

[B6] Brewster D. C. (1997). Current controversies in the management of aortoiliac occlusive disease. J. Vasc. Surg. 25, 365–379. 10.1016/s0741-5214(97)70359-8 9052572

[B7] Brewster L. P. Brey E. M. Greisler H. P. (2006). Cardiovascular gene delivery: The good road is awaiting. Adv. Drug Deliv. Rev. 58, 604–629. 10.1016/j.addr.2006.03.002 16769148 PMC3337725

[B8] Byzova T. V. Goldman C. K. Pampori N. Thomas K. A. Bett A. Shattil S. J. (2000). A mechanism for modulation of cellular responses to VEGF: Activation of the integrins. Mol. Cell 6, 851–860. 10.1016/s1097-2765(00)00083-6 11090623

[B9] Case J. Mead L. E. Bessler W. K. Prater D. White H. A. Saadatzadeh M. R. (2007). Human CD34+AC133+VEGFR-2+ cells are not endothelial progenitor cells but distinct, primitive hematopoietic progenitors. Exp. Hematol. 35, 1109–1118. 10.1016/j.exphem.2007.04.002 17588480

[B10] Chen T. T. Luque A. Lee S. Anderson S. M. Segura T. Iruela-Arispe M. L. (2010). Anchorage of VEGF to the extracellular matrix conveys differential signaling responses to endothelial cells. J. Cell Biol. 188, 595–609. 10.1083/jcb.200906044 20176926 PMC2828913

[B11] Chlupáč J. Filová E. Bačáková L. (2009). Blood vessel replacement: 50 years of development and tissue engineering paradigms in vascular surgery. Physiol. Res. 58 (2), S119–S140. 10.33549/physiolres.931918 20131930

[B12] Cooke J. P. Tsao P. S. (1994). Is NO an endogenous antiatherogenic molecule? Arterioscler. Thromb. J. Vasc. Biol. 14, 653–655. 10.1161/01.atv.14.5.653 8172841

[B13] Cornelius R. M. Sanchez J. Olsson P. Brash J. L. (2003). Interactions of antithrombin and proteins in the plasma contact activation system with immobilized functional heparin. J. Biomed. Mat. Res. A 67, 475–483. 10.1002/jbm.a.10118 14566788

[B14] de Mel A. Jell G. Stevens M. M. Seifalian A. M. (2008). Biofunctionalization of biomaterials for accelerated *in situ* endothelialization: A review. Biomacromolecules 9, 2969–2979. 10.1021/bm800681k 18831592

[B15] Deng J. Yuan S. Li X. Wang K. Xie L. Li N. (2017). Heparin/DNA aptamer co-assembled multifunctional catecholamine coating for EPC capture and improved hemocompatibility of vascular devices. Mat. Sci. Eng. C Mat. Biol. Appl. 79, 305–314. 10.1016/j.msec.2017.05.057 28629023

[B16] Donovan D. L. Schmidt S. P. Townshend S. P. Njus G. O. Sharp W. V. (1990). Material and structural characterization of human saphenous vein. J. Vasc. Surg. 12, 531–537. 10.1067/mva.1990.22707 2231964

[B17] Duan Y. Yu S. Xu P. Wang X. Feng X. Mao Z. (2019). Co-immobilization of CD133 antibodies, vascular endothelial growth factors, and REDV peptide promotes capture, proliferation, and differentiation of endothelial progenitor cells. Acta Biomater. 96, 137–148. 10.1016/j.actbio.2019.07.004 31284097

[B18] Dziemidowicz K. Sang Q. Wu J. Zhang Z. Zhou F. Lagaron J. M. (2021). Electrospinning for healthcare: Recent advancements. J. Mat. Chem. B 9, 939–951. 10.1039/d0tb02124e 33367446

[B19] Ercolani E. Del Gaudio C. Bianco A. (2015). Vascular tissue engineering of small-diameter blood vessels: Reviewing the electrospinning approach. J. Tissue Eng. Regen. Med. 9, 861–888. 10.1002/term.1697 23365048

[B20] Ferrara N. Gerber H.-P. LeCouter J. (2003). The biology of VEGF and its receptors. Nat. Med. 9, 669–676. 10.1038/nm0603-669 12778165

[B21] Green J. J. Elisseeff J. H. (2016). Mimicking biological functionality with polymers for biomedical applications. Nature 540, 386–394. 10.1038/nature21005 27974772 PMC8186828

[B22] Hahn C. Schwartz M. A. (2009). Mechanotransduction in vascular physiology and atherogenesis. Nat. Rev. Mol. Cell Biol. 10, 53–62. 10.1038/nrm2596 19197332 PMC2719300

[B23] Harskamp R. E. Lopes R. D. Baisden C. E. de Winter R. J. Alexander J. H. (2013). Saphenous vein graft failure after coronary artery bypass surgery: Pathophysiology, management, and future directions. Ann. Surg. 257, 824–833. 10.1097/SLA.0b013e318288c38d 23574989

[B24] Jesmer A. H. Wylie R. G. (2020). Controlling experimental parameters to improve characterization of biomaterial fouling. Front. Chem. 8, 604236. 10.3389/fchem.2020.604236 33363113 PMC7759637

[B25] Kang K. Choi I. S. Nam Y. (2011). A biofunctionalization scheme for neural interfaces using polydopamine polymer. Biomaterials 32, 6374–6380. 10.1016/j.biomaterials.2011.05.028 21652066

[B26] Khakoo A. Y. Finkel T. (2005). Endothelial progenitor cells. Annu. Rev. Med. 56, 79–101. 10.1146/annurev.med.56.090203.104149 15660503

[B27] Kolbe M. Dohle E. Katerla D. Kirkpatrick C. J. Fuchs S. (2010). Enrichment of outgrowth endothelial cells in high and low colony-forming cultures from peripheral blood progenitors. Tissue Eng. Part C Methods 16, 877–886. 10.1089/ten.TEC.2009.0492 19891540 PMC2953933

[B28] Ku S. H. Park C. B. (2010). Human endothelial cell growth on mussel-inspired nanofiber scaffold for vascular tissue engineering. Biomaterials 31, 9431–9437. 10.1016/j.biomaterials.2010.08.071 20880578

[B29] Kutikhin A. G. Tupikin A. E. Matveeva V. G. Shishkova D. K. Antonova L. V. Kabilov M. R. (2020). Human peripheral blood-derived endothelial colony-forming cells are highly similar to mature vascular endothelial cells yet demonstrate a transitional transcriptomic signature. Cells 9, E876. 10.3390/cells9040876 PMC7226818 32260159

[B30] Lee H. Dellatore S. M. Miller W. M. Messersmith P. B. (2007). Mussel-Inspired surface Chemistry for multifunctional coatings. Science 318, 426–430. 10.1126/science.1147241 17947576 PMC2601629

[B31] Lehtinen T. Kiviniemi T. O. Ylitalo A. Mikkelsson J. Airaksinen J. K. E. Karjalainen P. P. (2012). Early vascular healing after endothelial progenitor cell capturing stent implantation. J. Invasive Cardiol. 24, 631–635. 23220976

[B32] Li L. Liu H. Xu C. Deng M. Song M. Yu X. (2017). VEGF promotes endothelial progenitor cell differentiation and vascular repair through connexin 43. Stem Cell Res. Ther. 8, 237. 10.1186/s13287-017-0684-1 29065929 PMC5655878

[B33] Lu S. Zhang P. Sun X. Gong F. Yang S. Shen L. (2013). Synthetic ePTFE grafts coated with an anti-CD133 antibody-functionalized heparin/collagen multilayer with rapid *in vivo* endothelialization properties. ACS Appl. Mat. Interfaces 5, 7360–7369. 10.1021/am401706w 23859593

[B34] Mahara A. Kitagawa K. Otaka A. Nakaoki T. Ishihara K. Yamaoka T. (2021). Impact of REDV peptide density and its linker structure on the capture, movement, and adhesion of flowing endothelial progenitor cells in microfluidic devices. Mat. Sci. Eng. C Mat. Biol. Appl. 129, 112381. 10.1016/j.msec.2021.112381 34579900

[B35] Medina R. J. Barber C. L. Sabatier F. Dignat-George F. Melero-Martin J. M. Khosrotehrani K. (2017). Endothelial progenitors: A consensus statement on nomenclature. Stem Cells Transl. Med. 6, 1316–1320. 10.1002/sctm.16-0360 28296182 PMC5442722

[B36] Melero-Martin J. M. (2022). Human endothelial colony-forming cells. Cold Spring Harb. Perspect. Med. 12, a041154. 10.1101/cshperspect.a041154 35379656 PMC9530069

[B37] Mi H.-Y. Jing X. Li Z.-T. Lin Y.-J. Thomson J. A. Turng L.-S. (2019). Fabrication and modification of wavy multicomponent vascular grafts with biomimetic mechanical properties, antithrombogenicity, and enhanced endothelial cell affinity. J. Biomed. Mat. Res. B Appl. Biomater. 107, 2397–2408. 10.1002/jbm.b.34333 30689292

[B38] Moulisová V. Gonzalez-García C. Cantini M. Rodrigo-Navarro A. Weaver J. Costell M. (2017). Engineered microenvironments for synergistic VEGF - integrin signalling during vascularization. Biomaterials 126, 61–74. 10.1016/j.biomaterials.2017.02.024 28279265 PMC5354119

[B39] Mp L. Ja H. (2005). Synthetic biomaterials as instructive extracellular microenvironments for morphogenesis in tissue engineering. Nat. Biotechnol. 23, 47–55. 10.1038/nbt1055 15637621

[B40] Ochsenhirt S. E. Kokkoli E. McCarthy J. B. Tirrell M. (2006). Effect of RGD secondary structure and the synergy site PHSRN on cell adhesion, spreading and specific integrin engagement. Biomaterials 27, 3863–3874. 10.1016/j.biomaterials.2005.12.012 16563498

[B41] Pagani F. D. (2019). Commentary: Expanded polytetrafluoroethylene: Making the reoperative experience easier or making more reoperations? J. Thorac. Cardiovasc. Surg. 157, e263. 10.1016/j.jtcvs.2018.11.007 30503745

[B42] Pashneh-Tala S. MacNeil S. Claeyssens F. (2016). The tissue-engineered vascular graft-past, present, and future. Tissue Eng. Part B Rev. 22, 68–100. 10.1089/ten.teb.2015.0100 26447530 PMC4753638

[B43] Paterlini T. T. Nogueira L. F. B. Tovani C. B. Cruz M. A. E. Derradi R. Ramos A. P. (2017). The role played by modified bioinspired surfaces in interfacial properties of biomaterials. Biophys. Rev. 9, 683–698. 10.1007/s12551-017-0306-2 28831703 PMC5662046

[B44] Plouffe B. D. Njoka D. N. Harris J. Liao J. Horick N. K. Radisic M. (2007). Peptide-mediated selective adhesion of smooth muscle and endothelial cells in microfluidic shear flow. Langmuir ACS J. Surf. Colloids 23, 5050–5055. 10.1021/la0700220 17373836

[B45] Prezhdo O. V. Pereverzev Y. V. (2009). Theoretical aspects of the biological catch bond. Acc. Chem. Res. 42, 693–703. 10.1021/ar800202z 19331389

[B46] Qi P. Yan W. Yang Y. Li Y. Fan Y. Chen J. (2015). Immobilization of DNA aptamers via plasma polymerized allylamine film to construct an endothelial progenitor cell-capture surface. Colloids Surf. B Biointerfaces 126, 70–79. 10.1016/j.colsurfb.2014.12.001 25575347

[B47] Qin G. Ii M. Silver M. Wecker A. Bord E. Ma H. (2006). Functional disruption of α4 integrin mobilizes bone marrow–derived endothelial progenitors and augments ischemic neovascularization. J. Exp. Med. 203, 153–163. 10.1084/jem.20050459 16401693 PMC2118065

[B48] Radke D. Jia W. Sharma D. Fena K. Wang G. Goldman J. (2018). Tissue engineering at the blood-contacting surface: A review of challenges and strategies in vascular graft development. Adv. Healthc. Mat. 7, e1701461. 10.1002/adhm.201701461 PMC6105365 29732735

[B49] Reynolds M. M. Annich G. M. (2011). The artificial endothelium. Organogenesis 7, 42–49. 10.4161/org.7.1.14029 21289481 PMC3082033

[B50] Rodenberg E. J. Pavalko F. M. (2007). Peptides derived from fibronectin type III connecting segments promote endothelial cell adhesion but not platelet adhesion: Implications in tissue-engineered vascular grafts. Tissue Eng. 13, 2653–2666. 10.1089/ten.2007.0037 17883325

[B51] Rossi E. Poirault-Chassac S. Bieche I. Chocron R. Schnitzler A. Lokajczyk A. (2019). Human endothelial colony forming cells express intracellular CD133 that modulates their vasculogenic properties. Stem Cell Rev. Rep. 15, 590–600. 10.1007/s12015-019-09881-8 30879244

[B52] Sarkar S. Sales K. M. Hamilton G. Seifalian A. M. (2007). Addressing thrombogenicity in vascular graft construction. J. Biomed. Mat. Res. B Appl. Biomater. 82, 100–108. 10.1002/jbm.b.30710 17078085

[B53] Savio G. Rosso S. Meneghello R. Concheri G. (2018). Geometric modeling of cellular materials for additive manufacturing in biomedical field: A review. Appl. Bionics Biomech. 2018, 1–14. 10.1155/2018/1654782 PMC5816891 29487626

[B54] Seeto W. J. Tian Y. Lipke E. A. (2013). Peptide-grafted poly(ethylene glycol) hydrogels support dynamic adhesion of endothelial progenitor cells. Acta Biomater. 9, 8279–8289. 10.1016/j.actbio.2013.05.023 23770139

[B55] Song J. Chen Z. Murillo L. L. Tang D. Meng C. Zhong X. (2021). Hierarchical porous silk fibroin/poly(L-lactic acid) fibrous membranes towards vascular scaffolds. Int. J. Biol. Macromol. 166, 1111–1120. 10.1016/j.ijbiomac.2020.10.266 33159945

[B56] Su H. Xue G. Ye C. Wang Y. Zhao A. Huang N. (2017). The effect of anti-CD133/fucoidan bio-coatings on hemocompatibility and EPC capture. J. Biomater. Sci. Polym. Ed. 28, 2066–2081. 10.1080/09205063.2017.1373989 28854848

[B57] Tian Y. Seeto W. J. Páez-Arias M. A. Hahn M. S. Lipke E. A. (2022). Endothelial colony forming cell rolling and adhesion supported by peptide-grafted hydrogels. Acta Biomater. 152, 74–85. 10.1016/j.actbio.2022.08.047 36031035

[B58] Timmis A. Townsend N. Gale C. Grobbee R. Maniadakis N. Flather M. (2018), Esc. Sci. Doc. Group. European society of cardiology: Cardiovascular disease statistics 2017. Eur. Heart J. 39, 508–579. 10.1093/eurheartj/ehx628 29190377

[B59] Tyler B. Gullotti D. Mangraviti A. Utsuki T. Brem H. (2016). Polylactic acid (PLA) controlled delivery carriers for biomedical applications. Adv. Drug Deliv. Rev. 107, 163–175. 10.1016/j.addr.2016.06.018 27426411

[B60] Tzima E. Irani-Tehrani M. Kiosses W. B. Dejana E. Schultz D. A. Engelhardt B. (2005). A mechanosensory complex that mediates the endothelial cell response to fluid shear stress. Nature 437, 426–431. 10.1038/nature03952 16163360

[B61] van Beusekom H. M. M. Ertaş G. Sorop O. Serruys P. W. van der Giessen W. J. (2012). The Genous^TM^ endothelial progenitor cell capture stent accelerates stent re-endothelialization but does not affect intimal hyperplasia in porcine coronary arteries. Catheter. Cardiovasc. Interv. Off. J. Soc. Card. Angiogr. Interv. 79, 231–242. 10.1002/ccd.22928 21834062

[B62] Wei Y. Wang F. Guo Z. Zhao Q. (2022). Tissue-engineered vascular grafts and regeneration mechanisms. J. Mol. Cell. Cardiol. 165, 40–53. 10.1016/j.yjmcc.2021.12.010 34971664

[B63] Yang W. Zhang X. Wu K. Liu X. Jiao Y. Zhou C. (2016). Improving cytoactive of endothelial cell by introducing fibronectin to the surface of poly L-Lactic acid fiber mats via dopamine. Mat. Sci. Eng. C Mat. Biol. Appl. 69, 373–379. 10.1016/j.msec.2016.07.006 27612725

[B64] Ye L. Wu X. Duan H.-Y. Geng X. Chen B. Gu Y.-Q. (2012). The *in vitro* and *in vivo* biocompatibility evaluation of heparin-poly(ε-caprolactone) conjugate for vascular tissue engineering scaffolds. J. Biomed. Mat. Res. A 100, 3251–3258. 10.1002/jbm.a.34270 22733560

[B65] Zhao J. Wang W. Ye C. Li Y. You J. (2018). Gravity-driven ultrafast separation of water-in-oil emulsion by hierarchically porous electrospun Poly(L-lactide) fabrics. J. Membr. Sci. 563, 762–767. 10.1016/j.memsci.2018.06.053

