# Supplementary material for: A small-diameter vascular graft immobilized peptides for capturing endothelial colony-forming cells
Source: Front Bioeng Biotechnol. 2023 Apr 10;11:1154986. doi: 10.3389/fbioe.2023.1154986 (PMC10123284; doi:10.3389/fbioe.2023.1154986)
Supplement: Supplementary file 1 [file DataSheet1.docx]

Supplementary Material

**A Small-Diameter Vascular Scaffold Immobilized Peptides for Capturing Endothelial Colony-Forming Cells**

**Yaqi Tang^1†^,** **Lu Yin^2†^, Shuai Gao^1^,** **Xiaojing Long^3^,** **Zhanhui Du^1^, Yingchao Zhou^1^, Shuiyan Zhao^1^, Yue Cao^1^, Silin Pan^1*^**

^1^ Heart Center, Qingdao Women and Children’s Hospital, Qingdao University, Qingdao, Shandong, China.

^2^ College of Material, Chemistry and Chemical Engineering, Hangzhou Normal University, Hangzhou, China.

^3^ State Key Laboratory of Bio-fibers and Eco-textiles, Qingdao University, Qingdao, China.

**^†^**These authors contributed equally to this work

*** Correspondence:**

Silin Pan

silinpan@126.com

#
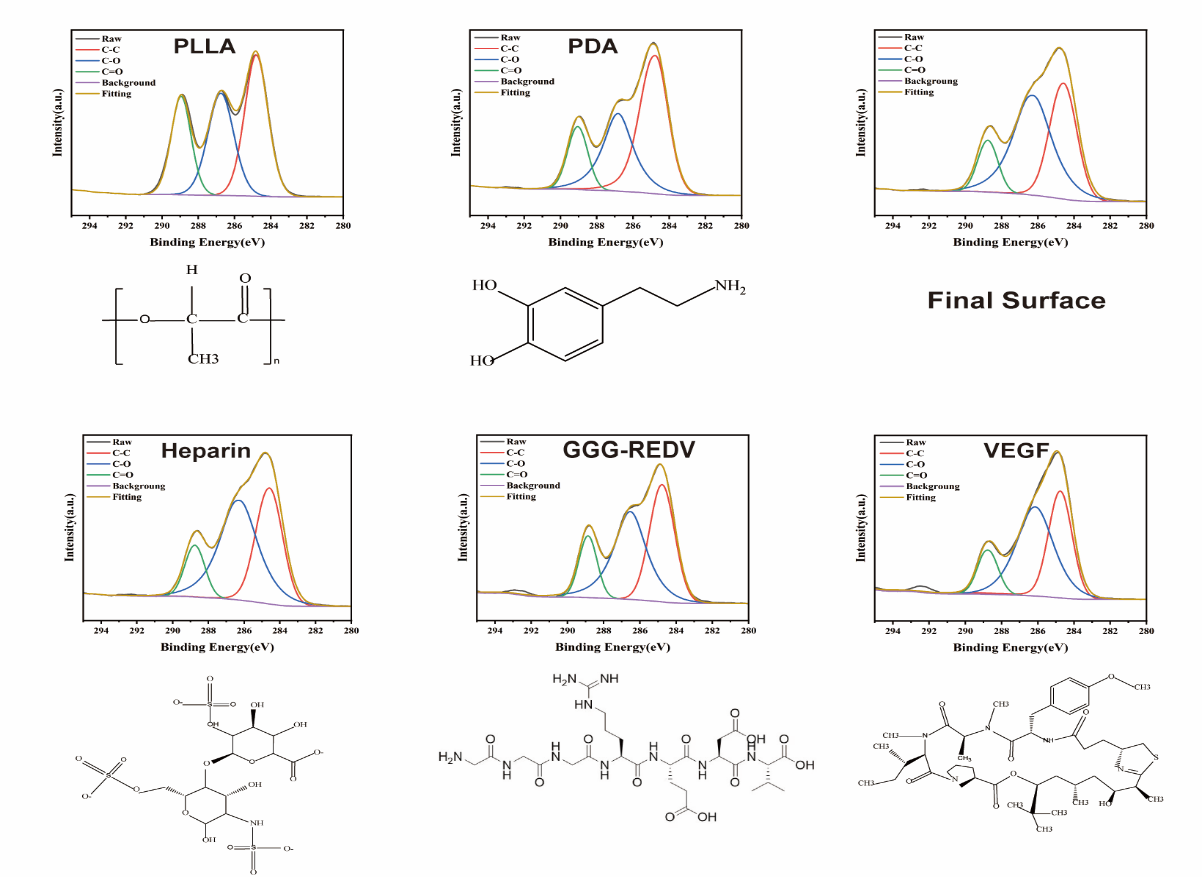
Supplementary Figures

**Figure S1.** The high-resolution C1s spectrum of various surfaces and chemical structure formula of surface coating molecules.


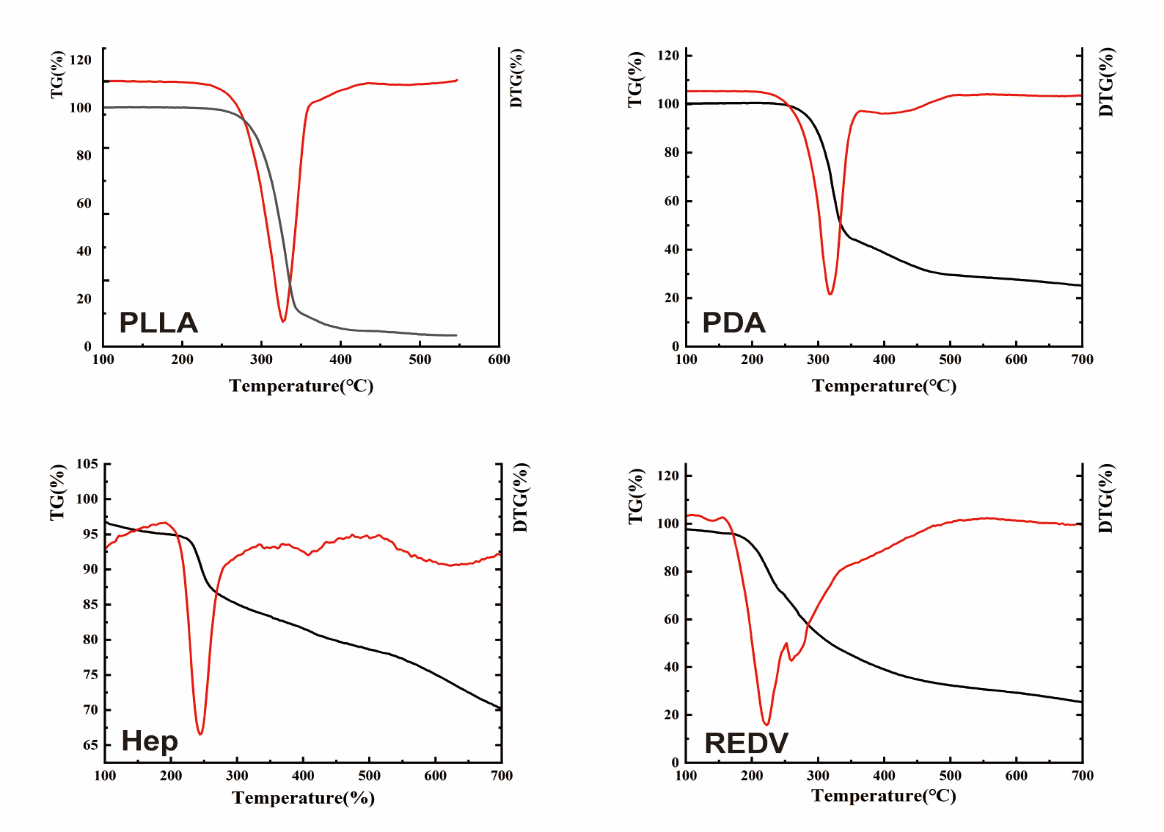


**Figure S2.** Thermal degradation temperature of each sample.

**
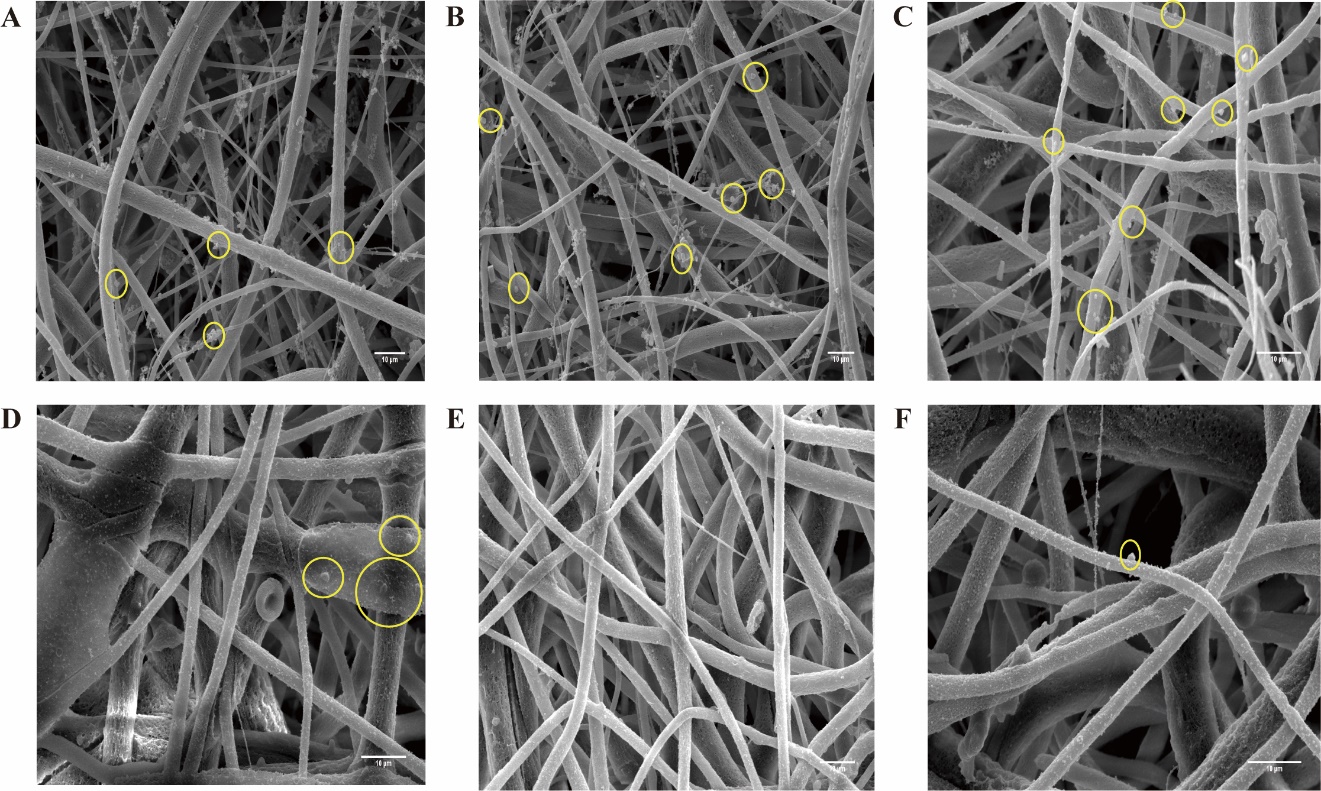
Figure S3.** The platelets adhesion on surfaces incubated with a different concentration ratio of PDA/Hep evaluated by SEM. (A) 1 mg/mL : 1 mg/mL (B) 1 mg/mL : 2 mg/mL (C) 1 mg/mL : 3 mg/mL (D) 2 mg/mL : 1 mg/mL (E) 2 mg/mL : 2 mg/mL (F) 2 mg/mL : 3 mg/mL


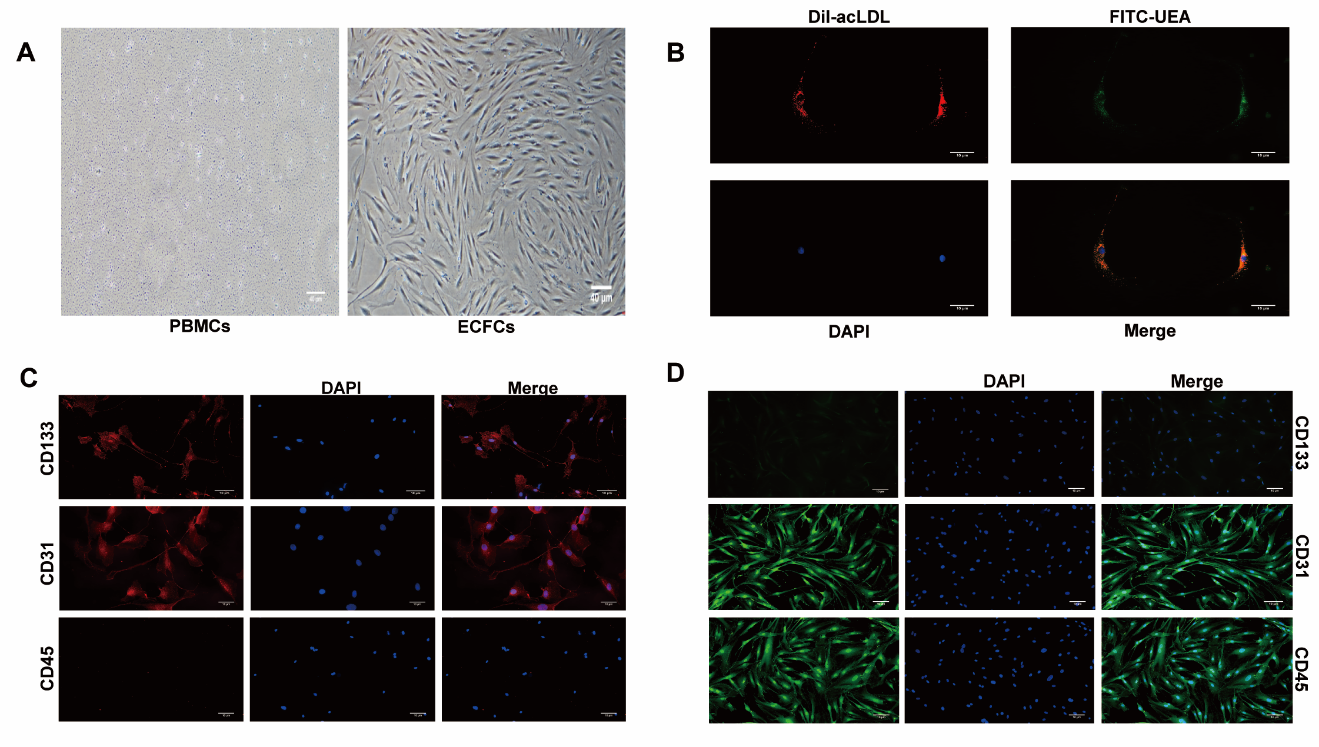
**Figure S4.** Extraction and cultivation of ECFC. (A) Isolated PBMCs from peripheral blood and differentiation to ECFCs after 11 days of culture. (B) ECFCs internalize acetylated low-density lipoprotein cholesterol (ac-LDL) and bind ulex europaeus agglutinin-1 (UEA-1). (C) ECFCs expressed the endothelial specification in terms of CD31, CD133 positive staining, and CD45-negative staining evaluated by IF. (D) Expression of CD133 in ECFCs decreased gradually while expression of CD 45 increased after 8 weeks of culture evaluated by IF.

**
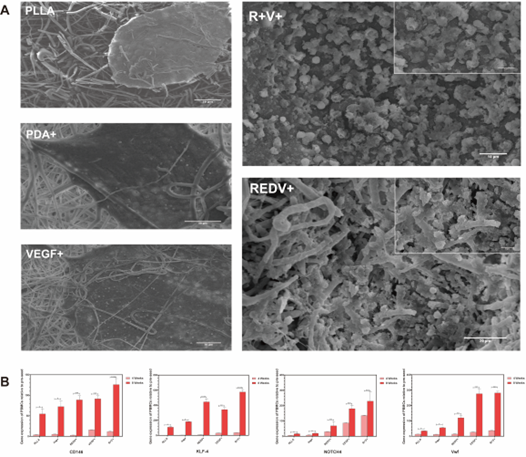
Figure S5.** Gene expression and growth morphology of PBMCs. (A) Morphology of PBMCs captured by various surfaces and cultures for 2 weeks evaluated by SEM. (B) Endothelial signature genes expression of PBMCs after captured on PLLA, Hep+, R+, V+, and R+V+ surfaces for 4 weeks and 8 weeks.

**Table S1. Primer sequences for RT-qPCR**

| **Gene** | **Primer Sequence** |
| --- | --- |
| GAPDH-R | ccgcctgcttcaccaccttcttg |
| GAPDH-F | gaacgggaagctcactggcatgg |
| vWF-R | gccatcccattccatctgcaggtc |
| vWF-F | gtgcgctgatgaccctgatgctg |
| NOTCH -R | cgtggaagggctgggactgctc |
| NOTCH -F | ccaggttctcgtcacagccctcg |
| CD146-R | cccgtgggactgctgaaggaagg |
| CD146-F | ggtgcttctgggcgggctcc |
| KLF4-R | ccccagctcagcaacggccac |
| KLF4-F | caggatcccggtggcatgagctc |
| KDR-R | tgtggaccgatgttgcctgtgag |
| KDR-F | tacccagccaaccgagcaggagag |
